# Supplementary material for: FIND Stroke Recovery Study (FIND): rationale and protocol for a longitudinal observational cohort study of trajectories of recovery and biomarkers poststroke
Source: BMJ Open. 2023 May 10;13(5):e072493. doi: 10.1136/bmjopen-2023-072493 (PMC10173956; doi:10.1136/bmjopen-2023-072493)
Supplement: Supplementary data [file bmjopen-2023-072493supp001.pdf]

Supplemental Table 1 Original protocol

Parameter	Details	Baseline	Follow-up 3, 6, 12 months, 2 and 5 years
Neurological impairment	NIHSS	X	X
Functional outcome	mRS	X	X
Cognitive function	MoCA, BNIS[a], Functional Ambulation Category, 15-Item Boston Naming Test*, Addenbrooke's Cognitive Examination-Revised test*	X	X
Motor function	FMA*	X	X
Cognitive function	Mesulam A and B, Trailmaking Test A, B and D[b], 10-word test from Repeatable Battery Mesulams test, modified Ashworth Scale for the Assessment of Neuropsychological Status[‡], FAS[‡c], Color-Word Interference test[‡], Letter-Number Sequencing Subtest from WAIS-VI[d],		X
Motor function	SAFE, Berg Balance Scale, 10m walking test	X	
Pre-stroke functional status, cognitive function, education and profession	SIS[e], IQCODE[f]	X	
Pre-stroke infection or major life event	Questionnaire	X	
Depression	HAD, PHQ-9[g], MADRS[h]		X
Fatigue	D-FIS		X
Sleepiness	ESS[i]		X
Quality of Life	Lisat 11[j]		X
Working and social situation	Questionnaire	X	X
Medical history, medication and rehab	Questionnaire	X	X
Cardiovascular risk factors	Questionnaire	X	X
Stroke-specific composite disability score	SIS[e]		X
Venous blood sampling	For protein biomarker profiling and extraction of genomic RNA	X	X

Stroke subtype	TOAST, OSCP and CSS	X

* If full function at baseline no further testing
ǂ Only patients <55 years at inclusion
[a] The Barrow Neurological Institute screen[1] for higher cerebral functions
[b] Trail making test D[2]
[c] Verbal Fluency semantic test FAS
[d] Letter-Number Sequencing Subtest from WAIS-VI[3]
[e] Stroke Impact Scale, full test, some domains still included (see methods section)
[f] Informant Questionnaire on Cognitive Decline in the Elderly[4]
[g] PHQ-9[5]
[h] Montgomery Åsberg Depression Rating Scale[6]
[i] Epworth Sleepiness Scale[7]
[j] Life Satisfaction Questionnaire[8]

REFERENCES

1. Prigatano GP. BNI Screen for higher cerebral functions: Rationale and initial validation. *BnI Quarterly*. 1991; 7: 2-9.
2. Llinàs-Reglà J, Vilalta-Franch J, López-Pousa S, Calvó-Perxas L, Torrents Rodas D and Garre-Olmo J. The Trail Making Test:Association With Other Neuropsychological Measures and Normative Values for Adults Aged 55 Years and Older From a Spanish-Speaking Population-Based Sample. *Assessment*. 2017; 24: 183-96.
3. Wechsler D. Wechsler adult intelligence scale–Fourth Edition (WAIS–IV). *San Antonio, TX: NCS Pearson*. 2008; 22: 1.
4. Jorm AF. The Informant Questionnaire on Cognitive Decline in the Elderly (IQCODE): a review. *International Psychogeriatrics*. 2004; 16: 275-93.
5. Kroenke K, Spitzer RL and Williams JB. The PHQ-9: validity of a brief depression severity measure. *Journal of general internal medicine*. 2001; 16: 606-13.
6. Montgomery SA and Asberg M. A new depression scale designed to be sensitive to change. *The British journal of psychiatry : the journal of mental science*. 1979; 134: 382-9.
7. Johns MW. A new method for measuring daytime sleepiness: the Epworth sleepiness scale. *Sleep*. 1991; 14: 540-5.
8. Fugl-Meyer AR, Bränholm I-B and Fugl-Meyer KS. Happiness and domain-specific life satisfaction in adult northern Swedes. *Clinical Rehabilitation*. 1991; 5: 25-33.
