# Supplementary material for: FIND Stroke Recovery Study (FIND): rationale and protocol for a longitudinal observational cohort study of trajectories of recovery and biomarkers poststroke
Source: BMJ Open. 2023 May 10;13(5):e072493. doi: 10.1136/bmjopen-2023-072493 (PMC10173956; doi:10.1136/bmjopen-2023-072493)
Supplement: Author's manuscript [file bmjopen-2023-072493.draft_revisions.pdf]

BMJ Open is committed to open peer review. As part of this commitment we make the peer review history of every article we publish publicly available.

When an article is published we post the peer reviewers' comments and the authors' responses online. We also post the versions of the paper that were used during peer review. These are the versions that the peer review comments apply to.

The versions of the paper that follow are the versions that were submitted during the peer review process. They are not the versions of record or the final published versions. They should not be cited or distributed as the published version of this manuscript.

BMJ Open is an open access journal and the full, final, typeset and author-corrected version of record of the manuscript is available on our site with no access controls, subscription charges or pay-per-view fees (http://bmjopen.bmj.com).

If you have any questions on BMJ Open's open peer review process please email info.bmjopen@bmj.com

BMJ Open

The FIND Stroke Recovery Study (FIND): Rationale and protocol for a longitudinal observational cohort study of trajectories of recovery and biomarkers post-stroke

Journal:	*BMJ Open*
Manuscript ID	bmjopen-2023-072493
Article Type:	Protocol
Date Submitted by the Author:	06-Feb-2023
Complete List of Authors:	Brännmark, Cecilia; Sahlgrenska Academy, Department of Laboratory Medicine, Institute of Biomedicine; Region of Västra Götaland, Department of Research, Development, Education and Innovation, Sahlgrenska University Hospital<br>Klasson, Sofia; Department of Laboratory Medicine, Institute of Biomedicine, the Sahlgrenska Academy, University of Gothenburg<br>Stanne, Tara; Institute of Biomedicine, Sahlgrenska Academy, University of Gothenburg., Department of Laboratory Medicine<br>Samuelsson, Hans; Institute of Psychology, Faculty of Social Sciences, University of Gothenburg; Region Västra Göraland, Sahlgrenska University Hospital, Department of Rehabilitation Medicin, Gothenburg, Sweden<br>Alt Murphy, Margit; Univ Gothenburg, Rehabilitation Medicine; Sahlgrenska Academy, Department of Clinical Neuroscience, Institute of Neuroscience and Physiology<br>Sunnerhagen, Katharina; Univ of Gothenburg, Inst of Neuroscience and Physiology<br>Åberg, N. David; Institute of Medicine, Sahlgrenska Academy, University of Gothenburg, Department of Internal Medicine; Region of Västra Götaland, Sahlgrenska University Hospital, Department of Acute Medicine and Geriatrics<br>Jalnefjord, Oscar; Sahlgrenska Academy, Department of Medical Radiation Sciences, Institute of Clinical Sciences; Region of Västra Götaland, Department of Medical Radiation Sciences, Institute of Clinical Sciences<br>Björkman-Burtscher, Isabella; Sahlgrenska Academy, Department of Radiology, Institute of Clinical Sciences; Region of Västra Götaland, Sahlgrenska University Hospital, Department of Radiology<br>Jood, Katarina; Region of Västra Götaland, Sahlgrenska University Hospital, Department of Neurology; Sahlgrenska Academy, Department of Clinical Neuroscience, Institute of Neuroscience and Physiology<br>Tatlisumak, Turgut; Sahlgrenska Academy, Department of Clinical Neuroscience, Institute of Neuroscience and Physiology; Region of Västra Götaland, Sahlgrenska University Hospital, Department of Neurology<br>Jern, Christina; Region of Västra Götaland, Department of Medical and Clinical Genetics; Sahlgrenska Academy, Department of Laboratory Medicine, Institute of Biomedicine
Keywords:	STROKE MEDICINE, Stroke < NEUROLOGY, Stroke medicine < INTERNAL

	MEDICINE

SCHOLARONE™
Manuscripts

The FIND Stroke Recovery Study (FIND): Rationale and protocol for a longitudinal observational cohort study of trajectories of recovery and biomarkers post-stroke

Cecilia Brännmark[1,2], M.D., PhD; Sofia Klasson[1], MScEng; Tara M Stanne[1], PhD; Hans Samuelsson[3,6], PhD; Margit Alt Murphy[4,5] PhD; Katharina Stibrant Sunnerhagen[6], M.D., PhD; David N. Åberg[7,8], MD, PhD; Oscar Jalnefjord[9,10], PhD; Isabella M. Björkman-Burtscher [11,12], M.D., PhD; Katarina Jood[4,13‡], M.D., PhD; Turgut Tatlisumak[4,13‡], M.D., PhD; Christina Jern[1,14‡], M.D., PhD

[1] Department of Laboratory Medicine, Institute of Biomedicine, the Sahlgrenska Academy, University of Gothenburg, Gothenburg, Sweden

[2] Region Västra Götaland, Sahlgrenska University Hospital, Department of Research, Development, Education and Innovation, Gothenburg, Sweden

[3] Institute of Psychology, Faculty of Social Sciences, University of Gothenburg, Gothenburg, Sweden

[4] Department of Clinical Neuroscience, Institute of Neuroscience and Physiology, the Sahlgrenska Academy, University of Gothenburg, Gothenburg, Sweden

[5] Region Västra Götaland, Sahlgrenska University Hospital, Department of Occupational Therapy and Physiotherapy, Gothenburg, Sweden

[6] Region Västra Göraland, Sahlgrenska University Hospital, Department of Rehabilitation Medicin, Gothenburg, Sweden

[7] Region Västra Götaland, Sahlgrenska University Hospital, Department of Acute Medicine and Geriatrics, Gothenburg, Sweden

[8]Department of Internal Medicine and Clinical Nutrition, Institute of Medicine, Sahlgrenska Academy, University of Gothenburg, Göteborg, Sweden

For peer review only

[9] Department of Medical Radiation Sciences, Institute of Clinical Sciences, Sahlgrenska Academy, University of Gothenburg, Gothenburg Sweden

[10] Region Västra Götaland, Sahlgrenska University Hospital, Department of Medical Physics and Biomedical Engineering, Gothenburg, Sweden

[11] Department of Radiology, Institute of Clinical Sciences, Sahlgrenska Academy, University of Gothenburg, Gothenburg Sweden

[12] Region Västra Götaland, Sahlgrenska University Hospital, Department of Radiology, Gothenburg, Sweden

[13] Region Västra Götaland, Sahlgrenska University Hospital, Department of Neurology, Gothenburg, Sweden

[14] Region Västra Götaland, Sahlgrenska University Hospital, Department of Clinical Genetics and Genomics, Gothenburg, Sweden

ǂ These authors contributed equally to this work.

Corresponding author: Cecilia Brännmark, PhD, Institute of Biomedicine, the Sahlgrenska Academy, University of Gothenburg, Box 440, SE-405 30 Gothenburg, Sweden. E-mail: cecilia.brannmark@vgregion.se

Short title: *FIND Stroke Recovery Study*

For peer review only

ABSTRACT

Introduction

Comprehensive studies mapping domain specific trajectories of recovery after stroke and biomarkers reflecting these processes are scarce. We therefore initiated an exploratory prospective observational study of stroke cases with repeated evaluation, called the *FIND Stroke Recovery Study* (*FIND*). We aim to capture trajectories of recovery from different impairments, including cognition, in combination with broad profiling of blood and imaging biomarkers of the recovery.

Methods and analysis

We recruit individuals with first-ever stroke at the stroke unit at the Sahlgrenska University Hospital, Sweden, to *FIND*. The inclusion started early 2018 and we aim to enrol minimum 500 patients until 2025. Neurological and cognitive impairments across multiple domains are assessed using validated clinical assessment methods, advanced neuroimaging is performed, and blood samples for biomarker measuring (protein, RNA, and DNA) at inclusion and follow-up visits at 3 months, 6 months, 1 year, 2 years, and 5 years post-stroke. At baseline and at each follow-up visit, we also register clinical variables known to influence outcomes such as pre-stroke functioning, stroke severity, acute interventions, rehabilitation, other treatments, socioeconomic status, infections (including Covid19), and other comorbidities. Recurrent vascular events are identified continuously in national health registers.

Ethics and dissemination

FIND composes a unique stroke cohort with detailed phenotyping, repetitive assessments of outcomes across multiple domains and patient reported outcomes as well as blood and imaging biomarker profiling. Ethical approval for the FIND study has been obtained from the Regional Ethics Review Board in Gothenburg. The results of this exploratory study will

For peer review only

provide novel data on the time course of recovery and biomarkers after stroke. The description of this protocol will inform the stroke research community of our ongoing study and facilitate comparisons with other data sets. The protocol is registered at http://www.clinicaltrials.gov, Study ID: NCT05708807.

Strengths and limitations of this study

+ Time-resolved data of post-stroke recovery in a sample of consecutively recruited and well-characterized stroke patients of all ages.
+ Follow-up includes detailed outcome metrics in multiple domains including motor, cognitive, and functional outcome after stroke.
+ Broad, repetitive biomarker profiling, in blood and using advanced brain imaging.
– Heterogeneity in the time point of sample collection in the acute phase.
– Persons suffering the most severe strokes will be underrepresented in the data, skewing results towards mild strokes.

For peer review only

INTRODUCTION AND RATIONALE

Stroke is the most common cause of adult disability[1] and the number of stroke survivors with disabilities is increasing[2]. Common stroke survivor disabilities include motor, cognitive, and visual impairments as well as depression, limitations in activities of daily living, restrictions in participation, and decreased quality of life. One of the first questions asked by newly diagnosed stroke patients' and their next of kin often concerns recovery and prognosis. Today that is not possible to answer as the knowledge on timelines, mechanisms, and individual variations, as well as predictors of recovery are not sufficient. On the group level, type of stroke, stroke severity, acute treatment, rehabilitation, depression, type 2 diabetes[3], hypertension, other comorbidities and living alone[4] are all factors that have been found to influence recovery after stroke. However, today knowledge on how to predict outcome and recovery after stroke on the individual level is limited as some of the variation cannot be explained by clinical parameters[5]. Also, the full range of mechanisms and factors leading to recovery, are not precisely known, and the molecular pathways that are involved in brain injury and repair in stroke are likely to vary over time[6]. For example, collateral circulation, reperfusion, and hemorrhagic transformation have key roles in the acute stage, while the determinants of neuroplasticity become important later[7].

The rate of recovery also differs substantially between affected domains and stroke survivors[7] and can be influenced by comorbidities and lifestyle. A majority of the existing studies conclude that most recovery occurs during the first 3-4 months after index stroke[8-11]. However, it is of note that these studies have focused on specific motor functions and global measurements of disability (e.g., modified Rankin Scale (mRS)) and do not capture the whole complexity of recovery after stroke and that there are data supporting domain-specific recovery patterns. For example, impairment in cognitive function is common during the early stage after stroke (up to 74%)[12] and successive improvement is described during the first six

For peer review only

months to a year[7, 9]. A recent review on recovery reported the largest improvement in cognitive function 61-180 days (or 2-6 months) after stroke onset[13]. Knowledge about the long-term trajectory after one year is more uncertain, a decline at group level has been suggested[14] but subgroups with no change or with recovery have also been described[15, 16]. Data suggests that for some impairments, such as aphasia and neglect, recovery may take place even years after index stroke[17].

Further studies of the long-term trajectory with sensitive measures of both global and domain-specific cognition and of possible mechanisms and modulators behind the change are warranted. Increased knowledge is important since it can be used to answer the questions on prognosis raised by patients and kin. Also, cognitive deficits after stroke are a major contributor to the burden of stroke and loss of quality of life. Moreover, we lack specific adjuvant treatments to enhance recovery after stroke. To establish such treatments increased knowledge on the pathways involved in stroke recovery and the trajectory of their activity is necessary, as has previously been suggested[18].

Developing blood biomarkers to monitor recovery trajectories and to assess when a certain treatment might benefit a certain patient would increase possibilities for developing new rehabilitation interventions after stroke and allow more individualized and precise treatment planning[19]. This knowledge can only be obtained by collecting a broad battery of clinical measures in combination with molecular profiling at set timepoints ranging from the acute phase to the chronic stages[7].

Here we aim to investigate the rate, pattern, and degree of recovery after stroke in various domains. We use validated clinical measures, including broad assessment of cognitive functioning as well as repeated neuroimaging and blood draws for analyses, in the acute phase and at 3, 6, and 12 months, as well as 2 and 5 years after stroke onset. In addition, we classify the etiological subtypes of all strokes.

For peer review only

METHODS AND ANALYSIS

Inclusion and exclusion criteria

We enrol patients presenting with first-ever ischemic stroke or intracerebral haemorrhage admitted to the stroke units at the Sahlgrenska University Hospital in Gothenburg, Sweden. Recruitment started January 18, 2018 and involved written informed consent as described below. We plan to include 500 stroke cases or more and estimate to enrol until 2025.

The inclusion criteria are:

i) ≥18 years of age; and

ii) first-ever acute ischemic stroke or intracerebral haemorrhage.

The exclusion criteria are:

i) pre-stroke mRS score of ≥3;

ii) severe neurodegenerative disease, cerebral neoplasm or terminal illness; and

iii) patients considered unlikely to be able to participate in or to understand and/or comply with study procedures during follow-up visits at the hospital.

For peer review only

Neuroimaging

During the hospital stay at the stroke unit all patients undergo computed tomography of the brain and if indicated magnetic resonance imaging (MRI) as part of the clinical routine. In case the MRI is not routinely included, all patients will be offered a brain MRI examination according to a study-specific protocol. The protocol includes morphological, functional, and microstructural imaging rendering structural, diffusion and perfusion metrics. From brain

MRI, imaging biomarkers can be extracted such as lesion location and size, tissue characteristics such as diffusion measures, and pre-stroke cerebral tissue condition including atrophy and white matter condition[20].

Patient and Public Involvement

Patients with living experience of stroke were involved in FIND during the design of the study. A survey with stroke survivors and representants from stroke patient association was utilised to identify which questions and areas were most important. Better prognostic information and cognitive deficits emerged as important areas. We hence designed our study to address those topics. Initially our ambition was to include a large number of metrics in order to comprehensively capture different outcomes (see supplementary Table 1 for original protocol). However, we found that such an extensive protocol left us with substantial missing data. After a new survey taking into account the respondent burden we chose to omit patient reported outcome measures (PROMs) capturing overlapping outcomes (see supplementary Table 1 for original protocol) in the final protocol.

Assessment protocol

The data collection First assessments started in February 2018, however, due to COVID-19–related restrictions, we had to modify the study protocol and pivot to remote testing via telephone during parts of 2020-21, in line with other studies[21].

At admission, the neurologic deficits are assessed using by the National Institutes of Health Stroke Scale (NIHSS; total score and scores for all subscales) and pre-stroke disability as by pre-stroke mRS. Patients are characterized regarding vascular risk factors, personal and

For peer review only

family medical history, social situation, education, employment, and lifestyle factors such as smoking and physical activity. Weight, height, and blood pressure are measured. We use selected domains from the patient-reported Stroke Impact Scale (SIS), a self-assessed measure: strength, memory and thinking, communication, mobility, and participation[22] and ask patients to report their pre-stroke status.

In addition to the NIHSS motor arm subscore, motor impairment of the upper extremity is assessed by trained physiotherapists (instructed by author MAM) using the Fugl–Meyer Assessment of Upper Extremity (FMA-UE) and Shoulder Abduction and Finger Extension (SAFE) score. Walking ability is assessed by functional Ambulation Category[23] and 10 m walking test and postural control by the Berg Balance Scale (BBS)[24]. Cognitive function is assessed by The Montreal Cognitive Assessment (MoCA)[25]. Participants with aphasia also do the 15-Item Boston Naming Test (BNT) items of language comprehension from Addenbrooke's Cognitive Examination-Revised test (ACE-R) and from Norsk grunntest for afasi (NGTA). A first-degree family history of stroke and TIA is obtained using a written questionnaire and structured interview. If a participant is unable to provide an adequate family history, a collateral history from a relative is sought. All tests performed can be overviewed in Figure 1. We also register clinical variables known to influence outcomes such as stroke severity and subtype, acute interventions, rehabilitation, other treatments, socioeconomic status, infections (including Covid19), and other comorbidities at inclusion and at all follow-up visits.

Surviving participants are invited to follow-up visits at the hospital at 3 and 6 months and 1, 2, and 5 years after the index stroke. At all visits, functional outcome is scored using the mRS, the degree of neurological impairments is assessed using NIHSS (total score and scores for all subscales), and cognitive outcome is evaluated using tests of global cognitive function (MoCA) and domain-specific functions of motor- and processing speed, visuospatial

For peer review only

attention, and executive function (Trailmaking Test A and B[26] and Random Shape Cancellation Test[27] ((RSCT) with (version A) and without (version B) 30 sec time restriction). Furthermore, all patients who underwent MRI at inclusion will be invited to a renewed MRI of the brain according to a study-specific protocol after 3 months. For study participants under 55 years of age we include extended testing of processing speed, selective attention, memory, and executive functions such as inhibition and verbal fluency, using part 1-3 of Color-Word Interference test (CWI) in Delis–Kaplan Executive Function System[28] (D-KEFS), the 10-word test from Repeatable Battery for the Assessment of Neuropsychological Status (RBANS) [29], and the Verbal Fluency Test[30] (FAS). For participants with aphasia at baseline, follow-ups with the 15-Item BNT[31] and tests of language comprehension were included. For participants with motor deficits at inclusion, follow-ups with FMA-UE, SAFE, functional Ambulation Category, 10 m walking test, BBS were included. All assessments are conducted by a specially trained research nurse. In Figure 1 all assessments and outcome metrics are listed, and time points are indicated.

We also gather PROMs including the selected domains of SIS described above as well as the SIS visual analogue scale on overall recovery, the Daily Fatigue Impact Scale (D-FIS)[32], a life satisfaction question on contentment with life in general from the Life Satisfactory Questionary[33], and the Hospital Anxiety and Depression Scale (HADS)[34] (Figure 1).

To ensure that individuals with the worse outcome/socioeconomic situations are not lost to follow up we collect the data in the following priority order: 1) patients receive questionnaires by mail and come to the hospital for tests, neuroimaging and blood draw; 2) patients receive mail questionnaires and a research nurse conducts a telephone interview; 3) patients receive mail questionnaires only; 4) when none of the above can be implemented, patients will be followed through national registers including Riksstroke[35], provided they have not chosen to

For peer review only

withdraw their participation.

Data retrieval

Data on deaths, and recurrent vascular events, and comorbidities over time during the whole study period are obtained from the Swedish Population Register, the National Patient Register, the Prescribed Drugs Register, and the National Register on Cause of Death as described[36]. For events in the Patient Register and the Cause of Death register we review medical records.

For patients who are unable to participate in our 3-month follow-up, data on outcomes are obtained from the national register Riksstroke[35], which has data on functional outcomes, participation and self-reported outcomes at 3 and 12 months after stroke. Data on health and social support are obtained from the National Board of Health and Welfare and data on education and employment status from the Longitudinal Database of Education, Income and Occupation Register (Swedish acronym: LISA for *Longitudinell integrationsdatabas för sjukförsäkrings- och arbetsmarknadsstudie*). Data on management of diabetes and hypertension are obtained from the National Diabetes Register and the Primary Care Registry, respectively. Data on prescribed drugs are obtained from the National Prescribed Drug Register.

Stroke subtyping

Ischemic stroke subtyping is performed by a trained physician using validated systems (Trial of Org. 10172 in Acute Stroke Treatment (TOAST) criteria[37], the Causative Classification of Stroke (CCS) system[38] and the Oxfordshire Community Stroke Project (OSCP)

For peer review only

classification[39] for ischemic stroke. Intracerebral bleedings (ICH) are classified using SMASH-U [40].

Blood sampling

A first blood sample is drawn as soon as possible after admission to the stroke unit, on a subgroup we also draw a sample 7 days after stroke. Thereafter blood samples are drawn at each follow-up visit. On all occasions, venous blood is collected after an overnight fast. Blood is drawn in several tubes and handled at room temperature, whole blood or supernatants, i.e., plasma and serum, are robot aliquoted within 90 minutes and stored in freezers at Biobank Väst, a part of the Swedish National Biobank, pending analysis, for details see (Table 1). Blood is also drawn for analyses of total leucocyte count and differential blood count at the Dept of Clinical Chemistry at our hospital on all occasions.

Table 1 Blood sample handling

Blood biomarker analysis

Clinically established biomarkers are commonly based on measurements of protein concentrations in plasma or serum, and we and others have previously found plasma proteins with subtype specific patterns[41, 42], plasma protein concentrations associated with recurrent vascular events[43, 44] as well as neurological and functional outcomes[44-47]. Here, planned analyses include multiomics profiling, on the metabolite, protein, RNA and DNA level, utilising the Swedish National infrastructure for state-of-the-art omics: Science for Life Laboratory (SciLifeLab), Uppsala and Bioinformatics and Data Centre, Gothenburg.

For peer review only

Type	Tube(s) and additive	Centrifugation	Storage
Whole blood	With 10%[α] ethylenediaminetetraacetic acid (EDTA)*	NA	-80 °C
EDTA plasma	With 10% [α] EDTA*	2000 g for 10 min	-80 °C
Citrate plasma	With 3.2% [α] natrium citrate*	2500 g for 20 min	-80 °C
Serum	Serum separator tube*	Left to coagulate 30-60 min and spun 2000 g for 10 min	-80 °C
Whole blood	Tempus™ Blood RNA[+]	NA	-20 °C

[α] Volume/volume
* Becton, Dickinson and Company, Becton Drive Franklin Lakes, NJ, USA
[+] Life Technologies, Carlsbad, CA, United States
NA, not applicable

For peer review only

STUDY AIMS

Primary objective

Determine temporal profiles describing the rate, pattern, and degree of changes in multiple domains of neurological, motor and cognitive functions with simultaneous profiling of changes in blood biomarker concentrations, in the acute and subacute phases and long-term of stroke. Determine individual and interindividual variations in recovery in the different domains.

Secondary objectives

i) Identification of clinical variables including neuroimaging predicting different neurological and cognitive outcomes in different domains and their recovery trajectories after stroke

ii) Stroke subtype-specific analysis of recovery trajectories

iii) Temporal profiles of biomarkers and biomarkers adding predictive value over and above clinical variables

iv) Determine trajectories of depression, fatigue, participation, and quality of life after stroke and their relation to other outcome measures.

Organization

The study is conducted with three principal investigators CJ, TT and KJ. The patients are recruited at the stroke-units at the Sahlgrenska University Hospital in Gothenburg. All follow-up invitations and visits are managed by research nurses. Data analysis will be overseen by the CJ and KJ research groups.

ETHICS AND DISSEMINATION

Ethical approval

This study was approved by the Regional Ethics Review Board in Gothenburg, and the Swedish Ethical Review Authority (REC number 187-17 and 2021-06071-02, respectively, Guarantor: CJ). Written informed consent is obtained from all participants prior to enrolment. For participants who are unable to communicate, consent is obtained from their next-of-kin. Participants may withdraw their consent at any time, and if so, the entire data of the participant will be cancelled, counted as missing.

Dissemmination

The protocol is registered at http://www.clinicaltrials.gov, Study ID: NCT05708807.

DISCUSSION

For peer review only

As stated in the Introduction, there is surprisingly little data on trajectories (i.e. repeated measures) of stroke recovery in several domains in itself and even less in combination with biomarkers. This protocol describes *FIND*, a unique longitudinal cohort study in stroke with detailed phenotyping and repetitive assessments of outcome measures across multiple domains. We use NIHSS and mRS, the most used measures of neurological deficits and functional outcome respectively, several motor function measures, and perform broad cognitive testing, essentially in accordance with recently published recommendations[48-50]. We also use several complementary outcome metrics, objectively tested as well as self-reported, to comprehensively capture a broad spectrum of outcomes including different dimensions according to the International Classification of Functioning, Disability and Health. In combination, we collect blood samples for broad biomarker profiling as well as advanced neuroimaging. The field of predictive biomarkers in ischemic stroke has not yet reached a conclusive state and even between high quality studies results are to some extent inconsistent[46]. Few ongoing studies have designs similar to FIND: e.g., OX-CHRONIC, STROKE-Cog, DISCOVERY[21, 51, 52] making external validation of our findings possible. In comparison to these, FIND is more focused on blood-based biomarkers. In line with our protocol, DISCOVERY[52] also include MRI biomarkers whereas OX-CHRONIC[21] and STROKE-Cog[51] do not.

Functional outcome is important for independence in stroke survivors. We supplement the most commonly used measure, mRS, with the finer grained NIHSS and several motor- and cognitive measures. We also include PROMs (e.g. domains of SIS) to complement our objective functionality measures. Moreover, as mRS is only moderately correlated to quality of life[53], we also include a question on life satisfaction.

Cognitive outcome after stroke is of great importance for the patients, their relatives, and care givers. Despite this, trajectories of cognitive recovery after stroke and mechanisms behind the

For peer review only

recovery are less studied than motor and other functionalities[54], likely due to the complexity of cognition. Thus, we chose to add several tests assessing similar domains to capture different aspects and nuances of cognition. We utilize the commonly used MoCA and add tests of different specific cognitive domains such as processing speed (e.g. TMT-A and CWT 1-2), attention (e.g. RSCT), executive functions (e.g. TMT-B and CWT 3), memory (10-word test from Repeatable Battery), and language and verbal fluency (FAS and BNT). To complement the cognitive tests, we also include PROMs such as domains of SIS, measuring patient reported impact of stroke in various domains of in daily life.

Stroke is associated with increased rates of depression and a previous study has found about one third of stroke patients to be affected by Post Stroke Depression[55]. A correlation between depressive symptoms (HADS-D >7) and performance in cognitive tests both on the global and domain specific level has been reported[56]. Also, depressive symptoms measured as HAD score >4 have been found to correlate to self-reported memory and thinking problems (using SIS)[57].

Because depression and cognitive impairment seem to be connected and reciprocally influential in stroke survivors, we are assessing mental health trajectories in our cohort. To enable comparison of our results with those from as many studies as possible we decided to include the widely used HADS which also includes anxiety.

Fatigue, a common symptom after stroke, can also add to cognitive dysfunction. Therefore, we included D-FIS to measure the impact of fatigue on cognitive recovery in this cohort. D-FIS is highly correlated to the more extensive Fatigue Impact Scale[58] but less time consuming for the patient.

The aetiology of ischemic stroke, classified according to TOAST[37], has as previously mentioned proven important for cognitive outcome[13], but also for other aspects of post-stroke

For peer review only

recovery such as persisting neurological deficits[59]. The study protocol presented here allows for TOAST subtyping of all ischemic strokes and subtype-specific analysis of outcome measures. In line with this we also have the possibility to classify the haemorrhagic stroke's aetiology into structural vascular lesions, medication, amyloid angiopathy, systemic disease, hypertension, or undetermined using the SMASH-U[40] classification system and to correlate the subtypes to biomarkers and outcome measures.

Acute imaging in stroke using CT and/or MRI aims primarily at identification of location and size of a lesion, exclusion of haemorrhage prior to treatment, differentiation between irreversibly affected brain tissue and tissue at risk, as well as identification of stenosis or occlusion in supplying vessels. We also perform neuroimaging with MRI at inclusion and perform detailed mapping of location and size of the lesion. Imaging can, however, also provide insights on pre-stroke status of the brain as well as poststroke tissue integrity within the lesion and in normal appearing cerebral tissue. Pre-existing disease markers, such as white matter lesions, atrophy, microinfarcts and co-morbidity of other aetiology, and post-stroke tissue integrity markers, such as metabolic, diffusion, and perfusion metrics, aide us to monitor disease progression and to target outcome prediction[20]. We also perform MRI at three time points post-stroke in order to assess for instance white matter condition.

Strengths and limitations

The strengths of the present study include time-resolved data of post-stroke recovery in a sample of consecutively recruited and well-characterized stroke patients of all ages. Moreover, the follow-up includes a comprehensive set of detailed outcome metrics in multiple domains. Taken together with our broad, repetitive biomarker profiling, in blood and using advanced MRI, this allows for exploration of biomarkers for cognitive, mood, and functional outcome as well as fatigue after stroke. Notably, this study is in accordance with guidelines for stroke outcome studies, that has been published after initiation of the present protocol[48].

For peer review only

Further strengths lie in the design of our protocol, which was influenced by a referendum with study participants, research nurses and representatives from the local stroke patient association[60].

Nonetheless, our study has important limitations. A limitation is heterogeneity in the time point of sample collection in the acute phase. For practical reasons it is not always possible to include participants withing 24 hours after symptom onset. We prioritize to be able to include as many study participants as possible and document the time of sampling to enable analysis of impact of the sampling time on the data. Another limitation, shared with most long-term follow-up studies after strokes, is that persons suffering the most severe strokes will be underrepresented in the data, skewing results towards mild strokes. This is due to both lower inclusion rate and higher likelihood for withdrawal from the study follow ups in severe stroke patients. To our advantage, we opt for telephone follow-ups when a physical visit is not possible. Moreover, very few study participants are fully lost as even though they will not come to follow-ups we can still follow them through national registers, such as the Swedish Stroke Register, the Prescribed Drug Register and the Cause of Death Register with consent from the participant. Also, due to the COVID-19 pandemic there will be a period of several months with lower inclusion rate and modified follow-up visits.

Significance/ Conclusion

The *FIND* study will provide an unprecedented opportunity to inform on the time course of recovery coupled to detailed biomarker analyses (imaging, multiomic profiling of protein, RNA and DNA) after stroke. The gained knowledge from this project will be helpful for designing future studies on biomarkers and interventions targeting stroke recovery. Moreover, the *FIND* study will provide an opportunity to find parts of the puzzle of extent and rate of

For peer review only

recovery for an individual. Hopefully, together with other studies in the field, it will lead to better information on prognosis to patients and their loved ones.

DECLARATIONS

Funding

This work is supported by the Swedish Research Council (2021-01114), the Swedish state under the agreement between the Swedish government and the county councils, the ALF-agreement (ALFGBG-965328, ALFGBG-965417, ALFGBG-966177, and ALFGBG-942664), the Swedish Heart and Lung Foundation (20220184), and the King Gustaf V:s and Queen Victoria's Freemasons' Foundation.

Contributorship statement

CB wrote the manuscript; SK, TMS, KSS, MAM, HS, IBB, OJ and DÅ contributed valuable expertise to study outline; KJ, TT and CJ conceived the study and are co-PIs of the study. All authors edited the manuscript and have approved the final version. CJ is the guarantor of the study.

Competing interests

Authors report no competing interests.

For peer review only

REFERENCES

1. Global, regional, and national burden of neurological disorders, 1990-2016: a systematic analysis for the Global Burden of Disease Study 2016. *Lancet Neurol*. 2019; 18: 459-80.
2. Collaborators GBDS. Global, regional, and national burden of stroke and its risk factors, 1990-2019: a systematic analysis for the Global Burden of Disease Study 2019. *Lancet Neurol*. 2021; 20: 795-820.
3. Alawieh A, Zhao J and Feng W. Factors affecting post-stroke motor recovery: Implications on neurotherapy after brain injury. *Behavioural brain research*. 2018; 340: 94-101.
4. Redfors P, Isaksén D, Lappas G, et al. Living alone predicts mortality in patients with ischemic stroke before 70 years of age: a long-term prospective follow-up study. *BMC neurology*. 2016; 16: 80.
5. Lindgren A and Maguire J. Stroke Recovery Genetics. *Stroke*. 2016; 47: 2427-34.
6. Lee JM, Fernandez-Cadenas I and Lindgren AG. Using Human Genetics to Understand Mechanisms in Ischemic Stroke Outcome: From Early Brain Injury to Long-Term Recovery. *Stroke*. 2021; 52: 3013-24.
7. Bernhardt J, Hayward KS, Kwakkel G, et al. Agreed definitions and a shared vision for new standards in stroke recovery research: The Stroke Recovery and Rehabilitation Roundtable taskforce. *International Journal of Stroke*. 2017; 12: 444-50.
8. Toschke AM, Tilling K, Cox AM, Rudd AG, Heuschmann PU and Wolfe CD. Patient-specific recovery patterns over time measured by dependence in activities of daily living after stroke and post-stroke care: the South London Stroke Register (SLSR). *Eur J Neurol*. 2010; 17: 219-25.
9. Lee KB, Lim SH, Kim KH, et al. Six-month functional recovery of stroke patients: a multi-time-point study. *International Journal of Rehabilitation Research*. 2015; 38: 173-80.
10. Verheyden G, Nieuwboer A, De Wit L, et al. Time Course of Trunk, Arm, Leg, and Functional Recovery After Ischemic Stroke. *Neurorehabilitation and Neural Repair*. 2008; 22: 173-9.
11. Chye A, Hackett ML, Hankey GJ, et al. Repeated Measures of Modified Rankin Scale Scores to Assess Functional Recovery From Stroke: AFFINITY Study Findings. *Journal of the American Heart Association*. 2022; 11: e025425.
12. Nys GM, van Zandvoort MJ, de Kort PL, Jansen BP, de Haan EH and Kappelle LJ. Cognitive disorders in acute stroke: prevalence and clinical determinants. *Cerebrovasc Dis*. 2007; 23: 408-16.
13. Saa JP, Tse T, Baum CM, et al. Cognitive Recovery After Stroke: A Meta-analysis and Metaregression of Intervention and Cohort Studies. *Neurorehabil Neural Repair*. 2021; 35: 585-600.
14. Lo JW, Crawford JD, Desmond DW, et al. Long-Term Cognitive Decline After Stroke: An Individual Participant Data Meta-Analysis. *Stroke*. 2022; 53: 1318-27.
15. Hochstenbach JB, den Otter R and Mulder TW. Cognitive recovery after stroke: a 2-year follow-up. *Arch Phys Med Rehabil*. 2003; 84: 1499-504.
16. Turunen KEA, Laari SPK, Kauranen TV, et al. Domain-Specific Cognitive Recovery after First-Ever Stroke: A 2-Year Follow-Up. *J Int Neuropsychol Soc*. 2018; 24: 117-27.
17. Patel M, Coshall C, Rudd AG and Wolfe CD. Natural history of cognitive impairment after stroke and factors associated with its recovery. *Clin Rehabil*. 2003; 17: 158-66.
18. Boyd LA, Hayward KS, Ward NS, et al. Biomarkers of Stroke Recovery: Consensus-Based Core Recommendations from the Stroke Recovery and Rehabilitation Roundtable. *Neurorehabil Neural Repair*. 2017; 31: 864-76.
19. Kamtchum-Tatuene J and Jickling GC. Blood Biomarkers for Stroke Diagnosis and Management. *Neuromolecular Med*. 2019; 21: 344-68.
20. Sagnier S and Sibon I. The new insights into human brain imaging after stroke. *Journal of neuroscience research*. 2022; 100: 1171-81.
21. Demeyere N, Williams OA, Milosevich E, et al. Long-term psychological consequences of stroke (OX-CHRONIC): A longitudinal study of cognition in relation to mood and fatigue after stroke: Protocol. *Eur Stroke J*. 2021; 6: 428-37.

For peer review only

22. Duncan PW, Bode RK, Min Lai S, Perera S and Glycine Antagonist in Neuroprotection Americans I. Rasch analysis of a new stroke-specific outcome scale: the Stroke Impact Scale. *Arch Phys Med Rehabil*. 2003; 84: 950-63.
23. Mehrholz J, Wagner K, Rutte K, Meissner D and Pohl M. Predictive validity and responsiveness of the functional ambulation category in hemiparetic patients after stroke. *Arch Phys Med Rehabil*. 2007; 88: 1314-9.
24. Berg KO, Wood-Dauphinee SL, Williams JI and Maki B. Measuring balance in the elderly: validation of an instrument. *Canadian journal of public health = Revue canadienne de sante publique*. 1992; 83 Suppl 2: S7-11.
25. Nasreddine ZS, Phillips NA, Bédirian V, et al. The Montreal Cognitive Assessment, MoCA: a brief screening tool for mild cognitive impairment. *J Am Geriatr Soc*. 2005; 53: 695-9.
26. Reitan RM. The relation of the trail making test to organic brain damage. *Journal of consulting psychology*. 1955; 19: 393-4.
27. Lowery N, Ragland JD, Gur RC, Gur RE and Moberg PJ. Normative data for the symbol cancellation test in young healthy adults. *Appl Neuropsychol*. 2004; 11: 218-21.
28. Delis D KE, Kramer J. . D-KEFS, Delis-Kaplan Executive Function System. *San Antonio, TX: The Psychological Corporation; 2001*. 2001.
29. Randolph C. *Repeatable Battery for the Assessment of Neuropsychological Status (RBANS)*. Psychological Corporation San Antonio, TX, 1998.
30. Strauss E SE, Spreen O. *A compendium of neuropsychological tests: administration, norms, and commentary, 3rd edn.*: Oxford, NY: Oxford University Press, 2006.
31. Mack WJ, Freed DM, Williams BW and Henderson VW. Boston Naming Test: shortened versions for use in Alzheimer's disease. *J Gerontol*. 1992; 47: P154-8.
32. Fisk JD and Doble SE. Construction and validation of a fatigue impact scale for daily administration (D-FIS). *Quality of Life Research*. 2002; 11: 263-72.
33. Fugl-Meyer AR, Bränholm I-B and Fugl-Meyer KS. Happiness and domain-specific life satisfaction in adult northern Swedes. *Clinical Rehabilitation*. 1991; 5: 25-33.
34. Zigmond AS and Snaith RP. The hospital anxiety and depression scale. *Acta psychiatrica Scandinavica*. 1983; 67: 361-70.
35. Riksstroke.org.2021. RIKSSTROKE The Swedish Stroke Register.
36. Jood K, Ladenvall C, Rosengren A, Blomstrand C and Jern C. Family history in ischemic stroke before 70 years of age: the Sahlgrenska Academy Study on Ischemic Stroke. *Stroke*. 2005; 36: 1383-7.
37. Adams HP, Jr., Bendixen BH, Kappelle LJ, et al. Classification of subtype of acute ischemic stroke. Definitions for use in a multicenter clinical trial. TOAST. Trial of Org 10172 in Acute Stroke Treatment. *Stroke*. 1993; 24: 35-41.
38. Ay H, Furie KL, Singhal A, Smith WS, Sorensen AG and Koroshetz WJ. An evidence-based causative classification system for acute ischemic stroke. *Ann Neurol*. 2005; 58: 688-97.
39. Bamford J, Sandercock P, Dennis M, Burn J and Warlow C. Classification and natural history of clinically identifiable subtypes of cerebral infarction. *Lancet (London, England)*. 1991; 337: 1521-6.
40. Meretoja A, Strbian D, Putaala J, et al. SMASH-U: a proposal for etiologic classification of intracerebral hemorrhage. *Stroke*. 2012; 43: 2592-7.
41. Stanne TM, Angerfors A, Andersson B, Brannmark C, Holmegaard L and Jern C. Longitudinal Study Reveals Long-Term Proinflammatory Proteomic Signature After Ischemic Stroke Across Subtypes. *Stroke*. 2022; 53: 2847-58.
42. Kang YH, Park MG, Noh KH, et al. Low serum TNF-related apoptosis-inducing ligand (TRAIL) levels are associated with acute ischemic stroke severity. *Atherosclerosis*. 2015; 240: 228-33.
43. Pedersen A, Redfors P, Lundberg L, et al. Haemostatic biomarkers are associated with long-term recurrent vascular events after ischaemic stroke. *Thromb Haemost*. 2016; 116: 537-43.
44. Bao Q, Zhang J, Wu X, et al. Clinical Significance of Plasma D-Dimer and Fibrinogen in Outcomes after Stroke: A Systematic Review and Meta-Analysis. *Cerebrovasc Dis*. 2022: 1-26.

For peer review only

45. Pedersen A, Stanne TM, Redfors P, et al. Fibrinogen concentrations predict long-term cognitive outcome in young ischemic stroke patients. *Res Pract Thromb Haemost*. 2018; 2: 339-46.
46. Montellano FA, Ungethüm K, Ramiro L, et al. Role of Blood-Based Biomarkers in Ischemic Stroke Prognosis: A Systematic Review. *Stroke*. 2021; 52: 543-51.
47. Lagging C, Klasson S, Pedersen A, et al. Investigation of 91 proteins implicated in neurobiological processes identifies multiple candidate plasma biomarkers of stroke outcome. *Sci Rep*. 2022; 12: 20080.
48. Lindgren AG, Braun RG, Juhl Majersik J, et al. International stroke genetics consortium recommendations for studies of genetics of stroke outcome and recovery. *International Journal of Stroke*. 2022; 17: 260-8.
49. Prange-Lasonder GB, Alt Murphy M, Lamers I, et al. European evidence-based recommendations for clinical assessment of upper limb in neurorehabilitation (CAULIN): data synthesis from systematic reviews, clinical practice guidelines and expert consensus. *J Neuroeng Rehabil*. 2021; 18: 162.
50. Kwakkel G, Lannin NA, Borschmann K, et al. Standardized Measurement of Sensorimotor Recovery in Stroke Trials: Consensus-Based Core Recommendations from the Stroke Recovery and Rehabilitation Roundtable. *Neurorehabil Neural Repair*. 2017; 31: 784-92.
51. Drag LL, Mlynash M, Nassar H, et al. A longitudinal study of the post-stroke immune response and cognitive functioning: the StrokeCog study protocol. *BMC neurology*. 2020; 20: 313.
52. Rost NS, Meschia JF, Gottesman R, Wruck L, Helmer K and Greenberg SM. Cognitive Impairment and Dementia After Stroke: Design and Rationale for the DISCOVERY Study. *Stroke*. 2021; 52: e499-e516.
53. Skoglund E, Westerlind E, Persson HC and Sunnerhagen KS. Self-perceived impact of stroke: A longitudinal comparison between one and five years post-stroke. *Journal of rehabilitation medicine*. 2019; 51: 660-4.
54. McDonald MW, Black SE, Copland DA, et al. Cognition in stroke rehabilitation and recovery research: Consensus-based core recommendations from the second Stroke Recovery and Rehabilitation Roundtable. *International Journal of Stroke*. 2019; 14: 774-82.
55. Hommel M, Carey L and Jaillard A. Depression: cognition relations after stroke. *International journal of stroke : official journal of the International Stroke Society*. 2015; 10: 893-6.
56. Williams OA and Demeyere N. Association of Depression and Anxiety With Cognitive Impairment 6 Months After Stroke. *Neurology*. 2021; 96: e1966-e74.
57. Ytterberg C, Cegrell L, von Koch L and Wiklander M. Depression symptoms 6 years after stroke are associated with higher perceived impact of stroke, limitations in ADL and restricted participation. *Sci Rep*. 2022; 12: 7816.
58. Pedersen A, Almkvist E, Holmegaard L, et al. Fatigue 7 years post-stroke: Predictors and correlated features. *Acta Neurol Scand*. 2022; 146: 295-303.
59. Pinto A, Tuttolomondo A, Di Raimondo D, Fernandez P and Licata G. Risk factors profile and clinical outcome of ischemic stroke patients admitted in a Department of Internal Medicine and classified by TOAST classification. *International angiology : a journal of the International Union of Angiology*. 2006; 25: 261-7.
60. Götaland SV. Strokeföreningen Västra Götaland.

For peer review only

Acknowledgements

The authors thank research nurses Susanne Nilsson, Ingrid Eriksson, Virginia Villarreal Calvo, Magdalena Hagelberg, and Matilda Errind Arvgård for their excellent work and assistance in recruitment of study participants and for conducting follow-up visits. We also thank the physiotherapists at the stroke unit performing the motor assessments. Furthermore, we thank the stroke survivors and their kin for generously donating their time to this study. In addition, we thank the Biobank Väst, Bioinformatics and Data Centre, Gothenburg, and SciLifeLab**.**

For peer review only

Figure legends

Figure 1 Overview of study protocol

* Performed at all visits except for inclusion. ** Only at inclusion. *** Not conducted at inclusion or at 6 months follow-up to prevent repetition bias. ¨ Performed at all visits except at 1 year.   Only performed at 3 months follow-up. $ for participants < 55 years old at index stroke. ø If aphasia at inclusion. ^ If deficits at inclusion. These fixed time points are complimented by register studies that constantly monitor recurrent stroke and other vascular events, medication, and deaths.

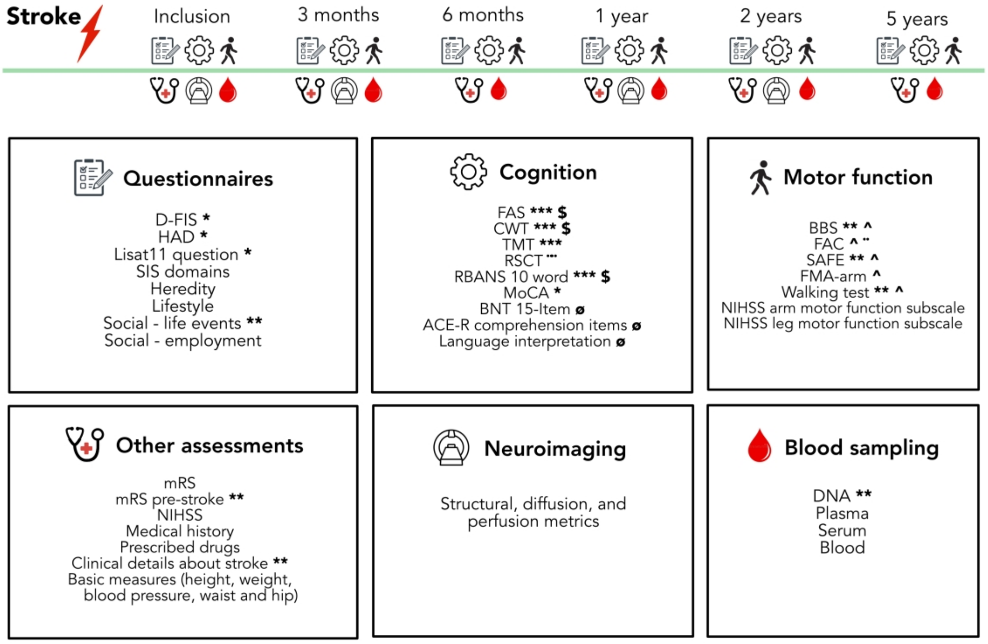

Figure 1 Overview of study protocol
* Performed at all visits except for inclusion. ** Only at inclusion. *** Not conducted at inclusion or at 6 months follow-up to prevent repetition bias. ¨ Performed at all visits except at 1 year. ⋯ Only performed at 3 months follow-up. $ for participants < 55 years old at index stroke. ø If aphasia at inclusion. ^ If deficits at inclusion. These fixed time points are complimented by register studies that constantly monitor recurrent stroke and other vascular events, medication, and deaths.

755x534mm (118 x 118 DPI)

Supplemental Table 1 Original protocol

Parameter	Details	Baseline	Follow-up 3, 6, 12 months, 2 and 5 years
Neurological impairment	NIHSS	X	X
Functional outcome	mRS	X	X
Cognitive function	MoCA, BNIS[a], Functional Ambulation Category, 15-Item Boston Naming Test*, Addenbrooke's Cognitive Examination-Revised test*	X	X
Motor function	FMA*	X	X
Cognitive function	Mesulam A and B, Trailmaking Test A, B and D[b], 10-word test from Repeatable Battery Mesulams test, modified Ashworth Scale for the Assessment of Neuropsychological Status[‡], FAS[‡c], Color-Word Interference test[‡], Letter-Number Sequencing Subtest from WAIS-VI[d],		X
Motor function	SAFE, Berg Balance Scale, 10m walking test	X	
Pre-stroke functional status, cognitive function, education and profession	SIS[e], IQCODE[f]	X	
Pre-stroke infection or major life event	Questionnaire	X	
Depression	HAD, PHQ-9[g], MADRS[h]		X
Fatigue	D-FIS		X
Sleepiness	ESS[i]		X
Quality of Life	Lisat 11[j]		X
Working and social situation	Questionnaire	X	X
Medical history, medication and rehab	Questionnaire	X	X
Cardiovascular risk factors	Questionnaire	X	X
Stroke-specific composite disability score	SIS[e]		X
Venous blood sampling	For protein biomarker profiling and extraction of genomic RNA	X	X

For peer review only

For peer review only - http://bmjopen.bmj.com/site/about/guidelines.xhtml

Stroke subtype	TOAST, OSCP and CSS	X

* If full function at baseline no further testing
ǂ Only patients <55 years at inclusion
[a] The Barrow Neurological Institute screen[1] for higher cerebral functions
[b] Trail making test D[2]
[c] Verbal Fluency semantic test FAS
[d] Letter-Number Sequencing Subtest from WAIS-VI[3]
[e] Stroke Impact Scale, full test, some domains still included (see methods section)
[f] Informant Questionnaire on Cognitive Decline in the Elderly[4]
[g] PHQ-9[5]
[h] Montgomery Åsberg Depression Rating Scale[6]
[i] Epworth Sleepiness Scale[7]
[j] Life Satisfaction Questionnaire[8]

REFERENCES

1. Prigatano GP. BNI Screen for higher cerebral functions: Rationale and initial validation. *BnI Quarterly*. 1991; 7: 2-9.
2. Llinàs-Reglà J, Vilalta-Franch J, López-Pousa S, Calvó-Perxas L, Torrents Rodas D and Garre-Olmo J. The Trail Making Test:Association With Other Neuropsychological Measures and Normative Values for Adults Aged 55 Years and Older From a Spanish-Speaking Population-Based Sample. *Assessment*. 2017; 24: 183-96.
3. Wechsler D. Wechsler adult intelligence scale–Fourth Edition (WAIS–IV). *San Antonio, TX: NCS Pearson*. 2008; 22: 1.
4. Jorm AF. The Informant Questionnaire on Cognitive Decline in the Elderly (IQCODE): a review. *International Psychogeriatrics*. 2004; 16: 275-93.
5. Kroenke K, Spitzer RL and Williams JB. The PHQ-9: validity of a brief depression severity measure. *Journal of general internal medicine*. 2001; 16: 606-13.
6. Montgomery SA and Asberg M. A new depression scale designed to be sensitive to change. *The British journal of psychiatry : the journal of mental science*. 1979; 134: 382-9.
7. Johns MW. A new method for measuring daytime sleepiness: the Epworth sleepiness scale. *Sleep*. 1991; 14: 540-5.
8. Fugl-Meyer AR, Bränholm I-B and Fugl-Meyer KS. Happiness and domain-specific life satisfaction in adult northern Swedes. *Clinical Rehabilitation*. 1991; 5: 25-33.

For peer review only

STROBE Statement—Checklist of items that should be included in reports of ***cohort studies***

	Item No	Recommendation	Page No
**Title and abstract**	1	(*a*) Indicate the study's design with a commonly used term in the title or the abstract	1
		(*b*) Provide in the abstract an informative and balanced summary of what was done and what was found	3-4
**Introduction**			
Background/rationale	2	Explain the scientific background and rationale for the investigation being reported	5-6
Objectives	3	State specific objectives, including any prespecified hypotheses	6
**Methods**			
Study design	4	Present key elements of study design early in the paper	6
Setting	5	Describe the setting, locations, and relevant dates, including periods of recruitment, exposure, follow-up, and data collection	6-7
Participants	6	(*a*) Give the eligibility criteria, and the sources and methods of selection of participants. Describe methods of follow-up	6
		(*b*) For matched studies, give matching criteria and number of exposed and unexposed	Not applicable
Variables	7	Clearly define all outcomes, exposures, predictors, potential confounders, and effect modifiers. Give diagnostic criteria, if applicable	12-13
Data sources/ measurement	8*	For each variable of interest, give sources of data and details of methods of assessment (measurement). Describe comparability of assessment methods if there is more than one group	7-12
Bias	9	Describe any efforts to address potential sources of bias	11
Study size	10	Explain how the study size was arrived at	Not applicable
Quantitative variables	11	Explain how quantitative variables were handled in the analyses. If applicable, describe which groupings were chosen and why	Not applicable
Statistical methods	12	(*a*) Describe all statistical methods, including those used to control for confounding	Not applicable
		(*b*) Describe any methods used to examine subgroups and interactions	
		(*c*) Explain how missing data were addressed	
		(*d*) If applicable, explain how loss to follow-up was addressed	
		(*e*) Describe any sensitivity analyses	
**Results**			
Participants	13*	(a) Report numbers of individuals at each stage of study—eg numbers potentially eligible, examined for eligibility, confirmed eligible, included in the study, completing follow-up, and analysed	Not applicable
		(b) Give reasons for non-participation at each stage	
		(c) Consider use of a flow diagram	
Descriptive data	14*	(a) Give characteristics of study participants (eg demographic, clinical, social) and information on exposures and potential confounders	Not applicable
		(b) Indicate number of participants with missing data for each variable of interest	
		(c) Summarise follow-up time (eg, average and total amount)	
Outcome data	15*	Report numbers of outcome events or summary measures over time	Not applicable

For peer review only - http://bmjopen.bmj.com/site/about/guidelines.xhtml

For peer review only

Main results	16	(*a*) Give unadjusted estimates and, if applicable, confounder-adjusted estimates and their precision (eg, 95% confidence interval). Make clear which confounders were adjusted for and why they were included<br>(*b*) Report category boundaries when continuous variables were categorized<br>(*c*) If relevant, consider translating estimates of relative risk into absolute risk for a meaningful time period	Not applicable
Other analyses	17	Report other analyses done—eg analyses of subgroups and interactions, and sensitivity analyses	Not applicable
**Discussion**			
Key results	18	Summarise key results with reference to study objectives	Not applicable
Limitations	19	Discuss limitations of the study, taking into account sources of potential bias or imprecision. Discuss both direction and magnitude of any potential bias	17-18
Interpretation	20	Give a cautious overall interpretation of results considering objectives, limitations, multiplicity of analyses, results from similar studies, and other relevant evidence	Not applicable
Generalisability	21	Discuss the generalisability (external validity) of the study results	Not applicable
**Other information**			
Funding	22	Give the source of funding and the role of the funders for the present study and, if applicable, for the original study on which the present article is based	22

*Give information separately for exposed and unexposed groups.

Note: An Explanation and Elaboration article discusses each checklist item and gives methodological background and published examples of transparent reporting. The STROBE checklist is best used in conjunction with this article (freely available on the Web sites of PLoS Medicine at http://www.plosmedicine.org/, Annals of Internal Medicine at http://www.annals.org/, and Epidemiology at http://www.epidem.com/). Information on the STROBE Initiative is available at http://www.strobe-statement.org.

For peer review only

BMJ Open

The FIND Stroke Recovery Study (FIND): Rationale and protocol for a longitudinal observational cohort study of trajectories of recovery and biomarkers post-stroke

Journal:	*BMJ Open*
Manuscript ID	bmjopen-2023-072493.R1
Article Type:	Protocol
Date Submitted by the Author:	18-Apr-2023

Complete List of Authors:	Brännmark, Cecilia; Sahlgrenska Academy, Department of Laboratory Medicine, Institute of Biomedicine; Region of Västra Götaland, Department of Research, Development, Education and Innovation, Sahlgrenska University Hospital<br>Klasson, Sofia; Department of Laboratory Medicine, Institute of Biomedicine, the Sahlgrenska Academy, University of Gothenburg<br>Stanne, Tara; Institute of Biomedicine, Sahlgrenska Academy, University of Gothenburg., Department of Laboratory Medicine; Region of Västra Götaland, Clinical Genetics and Biomedicine<br>Samuelsson, Hans; Institute of Psychology, Faculty of Social Sciences, University of Gothenburg; Region Västra Göraland, Sahlgrenska University Hospital, Department of Rehabilitation Medicin, Gothenburg, Sweden<br>Alt Murphy, Margit; Univ Gothenburg, Rehabilitation Medicine; Sahlgrenska Academy, Department of Clinical Neuroscience, Institute of Neuroscience and Physiology<br>Sunnerhagen, Katharina; Univ of Gothenburg, Inst of Neuroscience and Physiology<br>Åberg, N. David; Institute of Medicine, Sahlgrenska Academy, University of Gothenburg, Department of Internal Medicine; Region of Västra Götaland, Sahlgrenska University Hospital, Department of Acute Medicine and Geriatrics<br>Jalnefjord, Oscar; Sahlgrenska Academy, Department of Medical Radiation Sciences, Institute of Clinical Sciences; Region of Västra Götaland, Department of Medical Radiation Sciences, Institute of Clinical Sciences<br>Björkman-Burtscher, Isabella; Sahlgrenska Academy, Department of Radiology, Institute of Clinical Sciences; Region of Västra Götaland, Sahlgrenska University Hospital, Department of Radiology<br>Jood, Katarina; Region of Västra Götaland, Sahlgrenska University Hospital, Department of Neurology; Sahlgrenska Academy, Department of Clinical Neuroscience, Institute of Neuroscience and Physiology<br>Tatlisumak, Turgut; Sahlgrenska Academy, Department of Clinical Neuroscience, Institute of Neuroscience and Physiology; Region of Västra Götaland, Sahlgrenska University Hospital, Department of Neurology<br>Jern, Christina; Region of Västra Götaland, Department of Medical and Clinical Genetics; Sahlgrenska Academy, Department of Laboratory Medicine, Institute of Biomedicine

**Primary Subject Heading**:	Neurology
Secondary Subject Heading:	Rehabilitation medicine, Patient-centred medicine, Mental health, Cardiovascular medicine
Keywords:	STROKE MEDICINE, Stroke < NEUROLOGY, Stroke medicine < INTERNAL MEDICINE

SCHOLARONE™ Manuscripts

The FIND Stroke Recovery Study (FIND): Rationale and protocol for a longitudinal observational cohort study of trajectories of recovery and biomarkers post-stroke

Cecilia Brännmark[1,2], MD, PhD; Sofia Klasson[1], MScEng; Tara M Stanne[1,3], PhD; Hans Samuelsson[4,7], PhD; Margit Alt Murphy[5,6] PhD; Katharina Stibrant Sunnerhagen[7], MD, PhD; David N. Åberg[8,9], MD, PhD; Oscar Jalnefjord[10,11], PhD; Isabella M. Björkman-Burtscher[12,13], MD, PhD; Katarina Jood[5,14‡], MD, PhD; Turgut Tatlisumak[5,14‡], MD, PhD; Christina Jern[1,3‡], MD, PhD

[1]Department of Laboratory Medicine, Institute of Biomedicine, the Sahlgrenska Academy, University of Gothenburg

[2]Region Västra Götaland, Sahlgrenska University Hospital, Department of Research, Development, Education and Innovation

[3]Region Västra Götaland, Sahlgrenska University Hospital, Department of Clinical Genetics and Genomics

[4]Institute of Psychology, Faculty of Social Sciences, University of Gothenburg

[5]Department of Clinical Neuroscience, Institute of Neuroscience and Physiology, the Sahlgrenska Academy, University of Gothenburg

[6]Region Västra Götaland, Sahlgrenska University Hospital, Department of Occupational Therapy and Physiotherapy

[7]Region Västra Göraland, Sahlgrenska University Hospital, Department of Rehabilitation Medicin

[8]Region Västra Götaland, Sahlgrenska University Hospital, Department of Acute Medicine and Geriatrics

For peer review only

[9]Department of Internal Medicine and Clinical Nutrition, Institute of Medicine, Sahlgrenska Academy, University of Gothenburg

[10]Department of Medical Radiation Sciences, Institute of Clinical Sciences, Sahlgrenska Academy, University of Gothenburg

[11]Region Västra Götaland, Sahlgrenska University Hospital, Department of Medical Physics and Biomedical Engineering

[12]Department of Radiology, Institute of Clinical Sciences, Sahlgrenska Academy, University of Gothenburg

[13]Region Västra Götaland, Sahlgrenska University Hospital, Department of Radiology

[14]Region Västra Götaland, Sahlgrenska University Hospital, Department of Neurology

all Gothenburg, Sweden

ǂ These authors contributed equally to this work.

Corresponding author: Cecilia Brännmark, PhD, Institute of Biomedicine, University of Gothenburg, Box 440, SE-405 30 Gothenburg, Sweden. E-mail: cecilia.brannmark@vgregion.se

Short title: *FIND Stroke Recovery Study*

For peer review only

ABSTRACT

Introduction

Comprehensive studies mapping domain specific trajectories of recovery after stroke and biomarkers reflecting these processes are scarce. We therefore initiated an exploratory prospective observational study of stroke cases with repeated evaluation, the *FIND Stroke Recovery Study*. We aim to capture trajectories of recovery from different impairments, including cognition, in combination with broad profiling of blood and imaging biomarkers of the recovery.

Methods and analysis

We recruit individuals with first-ever stroke at the stroke unit at the Sahlgrenska University Hospital, Sweden, to *FIND*. The inclusion started early 2018 and we aim to enrol minimum 500 patients. Neurological and cognitive impairments across multiple domains are assessed using validated clinical assessment methods, advanced neuroimaging is performed, and blood samples for biomarker measuring (protein, RNA, and DNA) at inclusion and follow-up visits at 3 months, 6 months, 1 year, 2 years, and 5 years post-stroke. At baseline and at each follow-up visit, we also register clinical variables known to influence outcomes such as pre-stroke functioning, stroke severity, acute interventions, rehabilitation, other treatments, socioeconomic status, infections (including Covid19), and other comorbidities. Recurrent stroke and other major vascular events are identified continuously in national health registers.

Ethics and dissemination

FIND composes a unique stroke cohort with detailed phenotyping, repetitive assessments of outcomes across multiple neurological and cognitive domains and patient reported outcomes as well as blood and imaging biomarker profiling. Ethical approval for the FIND study has been obtained from the Regional Ethics Review Board in Gothenburg and the Swedish Ethics

For peer review only

Review Board. The results of this exploratory study will provide novel data on the time course of recovery and biomarkers after stroke. The description of this protocol will inform the stroke research community of our ongoing study and facilitate comparisons with other data sets. The protocol is registered at http://www.clinicaltrials.gov, Study ID: NCT05708807.

Strengths and limitations of this study

- + Time-resolved data of post-stroke recovery in a sample of consecutively recruited and well-characterized stroke patients of all ages.
- + Follow-up includes detailed outcome metrics in multiple domains including motor, cognitive, and functional outcome after stroke.
- + Broad, repetitive biomarker profiling, in blood and using advanced brain imaging.
- – Heterogeneity in the time point of sample collection in the acute phase.
- – Persons suffering the most severe strokes are likely to be underrepresented in the data, skewing results towards mild strokes.

For peer review only

INTRODUCTION AND RATIONALE

Stroke is the most common cause of adult disability[1] and the number of stroke survivors with disabilities is increasing[2]. Common stroke survivor disabilities include motor, cognitive, and visual impairments as well as depression, limitations in activities of daily living, restrictions in participation, and decreased quality of life. One of the first questions asked by newly diagnosed stroke patients' and their next of kin often concerns recovery and prognosis. Today that is not possible to answer as the knowledge on timelines, mechanisms, and individual variations, as well as predictors of recovery are not sufficient. On the group level, type of stroke, stroke size and location of brain lesion, acute treatment, rehabilitation, depression, type 2 diabetes, hypertension, other comorbidities and living alone[3, 4] are all factors that have been found to influence recovery after stroke. However, today knowledge on how to predict outcome and recovery after stroke on the individual level is limited as some of the variation cannot be explained by clinical parameters[5]. Also, the full range of mechanisms and factors leading to recovery, are not precisely known, and the molecular pathways that are involved in brain injury and repair in stroke are likely to vary over time[6]. For example, collateral circulation, reperfusion, and hemorrhagic transformation have key roles in the acute stage, while the determinants of neuroplasticity become important later[7].

The rate of recovery also differs substantially between type of impairment and stroke survivors[7] and can be influenced by comorbidities and lifestyle. A majority of the existing studies conclude that most recovery occurs during the first 3-4 months after index stroke[8-11]. However, it is of note that these studies have focused on specific motor functions and global measurements of disability (e.g., modified Rankin Scale (mRS)) and do not capture the whole complexity of recovery after stroke and that there are data supporting domain-specific recovery patterns. For example, impairment in cognitive function is common during the early stage after stroke (in at least 75%)[12-14] and successive improvement is described during the

For peer review only

first six months to a year[7, 9]. A recent review on recovery reported the largest improvement in cognitive function 61-180 days (or 2-6 months) after stroke onset[15]. Knowledge about the long-term trajectory after one year is more uncertain, some report stability in cognitive function between 3 months and 2 years after stroke for the majority of cases[16], while others found a decline at the group level[17]. There are also studies reporting that while some stroke survivors display no change in cognition or a decline, there are also stroke survivors who have improved cognitive function at long term follow-ups[18-20]. Data suggests that for some cognitive impairments, such as aphasia and neglect, recovery may take place even years after index stroke[21].

Further studies of the long-term trajectories with sensitive measures of both global and specific domains of cognition and of possible mechanisms and modulators behind the change are warranted. In addition to the FIND study presented here, there are some additional ongoing studies on this topic such as OX-CHRONIC, STROKE-Cog, DISCOVERY, ODYSSEY, R4VaD and APPLE[22-27], all with different focuses regarding age groups and research questions. Increased knowledge in this field is important since it can be used to answer the questions on prognosis raised by patients and kin. Also, cognitive deficits after stroke are a major contributor to the burden of stroke and loss of quality of life. Moreover, we lack specific adjuvant treatments to enhance recovery after stroke. To establish such treatments increased knowledge on the pathways involved in stroke recovery and the trajectory of their activity is necessary, as has previously been suggested[28].

Developing blood biomarkers to monitor recovery trajectories and to assess when a certain treatment might benefit a certain patient would increase possibilities for developing new rehabilitation interventions after stroke and allow more individualized and precise treatment planning[29]. This knowledge can only be obtained by collecting a broad battery of clinical

For peer review only

measures in combination with molecular profiling at set timepoints ranging from the acute phase to the chronic stages[7].

Here we aim to investigate the rate, pattern, and degree of recovery after stroke in various domains. We use validated clinical measures, including broad assessment of cognitive functioning as well as repeated neuroimaging and blood draws for analyses, in the acute phase and at 3, 6, and 12 months, as well as 2 and 5 years after stroke onset. In addition, we classify the etiological subtypes of all strokes.

METHODS AND ANALYSIS

Inclusion and exclusion criteria

We enroll patients presenting with first-ever ischemic stroke or intracerebral haemorrhage admitted to the stroke units at the Sahlgrenska University Hospital in Gothenburg, Sweden. Recruitment started January 2018 and involved written informed consent as described below. We plan to include 500 stroke cases or more, and we estimate to enroll until 2026 with follow-ups ongoing until all participants have had their 5-year follow-up (i.e. 2030). During the Covid-19 pandemic, study participants had to be followed up remotely. We therefore aim to have at least 300 participants who have been able to come to ≥3 in person follow-up visits. We will keep track of attrition with regards to participation rates in study visits and adjust the time line for inclusion accordingly. By the end of February 2023, 305 stroke cases had been enrolled and 5 cases had been at their last 5-year follow-up visit.

The inclusion criteria are:

i) ≥18 years of age; and

ii) first-ever acute ischemic stroke or intracerebral haemorrhage.

For peer review only

The exclusion criteria are:

i) pre-stroke mRS score of ≥3;

ii) severe neurodegenerative disease, cerebral neoplasm or terminal illness; and patients considered unlikely to be able to participate in study procedures during follow-up visits. However, we invite study participants with severe cognitive impairments and/or communication difficulties, because these persons can often participate fully or in parts of the study procedures with assistance of a next-of-kin, friend, or caregiver.

Patient and Public Involvement

Patients with living experience of stroke were involved in FIND during the design of the study. A survey with stroke survivors and representatives from stroke patient association was utilised to identify which questions and areas that were most important. Better prognostic information and cognitive deficits emerged as important areas. We hence designed our study to address those topics. Initially our ambition was to include a large number of metrics in order to comprehensively capture different outcomes (see supplementary Table 1 for original protocol). However, we found that such an extensive protocol left us with substantial missing data. After a new survey taking into account the respondent burden, we chose to omit some cognitive tests and a few patient reported outcome measures (PROMs) capturing overlapping outcomes in the final protocol.

Assessment protocol

Study inclusion started in January 2018, however, due to Covid-19 –related restrictions, we had to modify the study protocol and pivot to remote testing via telephone during parts of

For peer review only

2020-21, in line with other studies[22]. We then used a shorter version of the Montreal Cognitive Assessment (MoCA) which has been validated for this setting[30-32].

Assessments at baseline during the hospital stay at the stroke unit

The neurologic deficits at admission are assessed using by the National Institutes of Health Stroke Scale (NIHSS; total score and scores for all subscales). Pre-stroke disability is assessed by pre-stroke mRS. We also assessed perceived health status 4 weeks pre-stroke using the Stroke Impact Scale (SIS), and here we used the subscales for strength, memory and thinking, communication, mobility, and participation.

Patients are characterized regarding vascular risk factors, personal medical history including comorbidities, social situation, education, employment, and lifestyle factors such as smoking and physical activity. A first-degree family history of stroke and transient ischemic attacks and other cardiovascular diseases is obtained using a written questionnaire and structured interview. If a participant is unable to provide an adequate family history, a collateral history from a relative is sought. Weight, height, and blood pressure are measured. We register acute interventions, rehabilitation, other treatments, and complications such as infections (including Covid-19) during hospital stay. For patients treated with recanalization, i.e. thrombectomy and/or thrombolysis, we register the NIHSS score after this treatment.

In addition to the NIHSS motor arm subscore, motor impairment of the upper extremity is assessed by trained physiotherapists (instructed by author MAM) using the Fugl–Meyer Assessment of Upper Extremity (FMA-UE) and Shoulder Abduction and Finger Extension (SAFE) score. Walking ability is assessed by functional Ambulation Category[33] and 10 m walking test and postural control by the Berg Balance Scale (BBS)[34]. Global cognitive function is assessed by MoCA[35]. In addition, we use the following specific subtasks from MoCA: (1) Attention/Working memory (target detection, serial sevens, and digit forward and

For peer review only

backward); (2) Executive function (Trails B task, phonemic fluency, and verbal abstraction); (3) Visuospatial construction (three-dimensional figure copy and clock drawing); (4) Episodic memory (5-word recall); and (5) Language (naming task and sentence repetition). Participants with aphasia also perform the 15-Item Boston Naming test (BNT) and items of language comprehension from Addenbrooke's Cognitive Examination-Revised test (ACE-R) and from "Norsk grunntest for afasi" (NGTA). Participants are encouraged to complete as much of the tests as possible, and if completion is not possible the cause is noted to allow for analysis of possible confounders such as aphasia giving false low scores in cognitive tests of other domains.

Neuroimaging

During the hospital stay at the stroke unit all patients undergo computed tomography of the brain and if indicated magnetic resonance imaging (MRI) as part of the clinical routine. In case the MRI is not routinely included, all patients will be offered a brain MRI examination according to a study-specific protocol. The protocol includes morphological, functional, and microstructural imaging rendering structural, diffusion and perfusion metrics. From brain MRI, we will extract different imaging biomarkers such as lesion location and size, tissue characteristics such as diffusion measures, and pre-stroke cerebral tissue condition including atrophy and white matter condition[36].

Assessments at follow-up visits

Surviving participants are invited to follow-up visits at the hospital at 3 and 6 months and 1, 2, and 5 years after the index stroke (Fig 1 and cognitive tests are detailed in Table 1). At all visits, functional outcome is scored using the mRS, the degree of neurological impairments is

For peer review only

assessed using NIHSS (total score and scores for all subscales), and cognitive outcome is evaluated using the same tests as described above for the acute stage. In addition tests of attention, neglect, processing speed, executive functions, basic perception and motor speed are used such as the Trailmaking Test A, B[37] and D and Random Shape Cancellation test[38] (RSCT) with and without 30 sec time restriction (version A and B). For study participants under 55 years of age at baseline, we include extended testing of processing speed, selective attention, memory, and executive functions such as inhibition and verbal fluency using part 1-3 of Color-Word Interference test (CWI) in Delis–Kaplan Executive Function System (D-KEFS)[39], the 10-word test from Repeatable Battery for the Assessment of Neuropsychological Status (RBANS)[40], and the Verbal Fluency Test (FAS)[41]. Participants with aphasia also perform the 15-Item Boston Naming test (BNT)[42] and items of language comprehension from Addenbrooke's Cognitive Examination-Revised test (ACE-R) and from “Norsk grunntest for afasi” (NGTA).

Table 1 Cognitive tests in the *FIND* study

Time points	Tested cognitive domain	Tests
At all assessments	**Global cognitive function**	MoCA total score
	**Specific cognitive domains**	
At all assessments	Executive functions	MoCA-short trails B task, phonemic fluency, and verbal abstraction
	Attention/Working memory	MoCA-target detection, serial sevens, and digit forward and backward;
	Visuospatial construction	MoCA-three-dimensional figure copy and clock drawing
	Episodic memory	MoCA -5-word recall
	Language	MoCA -naming task and sentence repetition
At all assessments, for patients with aphasia at acute stage	Language - Aphasia	15-Item Boston Naming Test; language comprehension from Addenbrooke's Cognitive Examination-Revised test and from Norsk grunntest for afasi
At all follow-ups	Basic perception and motor speed	Trail making test-D (connecting empty circles by following a predetermined dotted track as fast as possible)
	Processing speed	Random Shape Cancellation Test with time restriction (RSCT-version B)

For peer review only

	Visuospatial attention	Total score of Random Shape Cancellation Test without time restriction (RSCT-version A)
	Attention - Neglect	Asymmetry score of Random Shape Cancellation Test without time restriction (RSCT-version A)
At follow-up 3m, 1y, 2y and 5y	Processing speed	Trail making test A
	Executive functions	Trail making test B
**Extended testing participants <55y**		
At follow-up 3m, 1y, 2y and 5y	Processing speed	Part 1 and 2 from Color-Word Interference test (CWI) in Delis–Kaplan Executive Function System (D-KEFS)
	Memory	10-word test from Repeatable Battery for the Assessment of Neuropsychological Status (RBANS)
	Executive functions - Selective attention and inhibition	The Inhibition part in Color-Word Interference test in D-KEFS
	Executive functions - fluency	The Verbal Fluency test (FAS)

For participants with motor deficits at inclusion, follow-ups with FMA-UE, SAFE, functional Ambulation Category, 10 m walking test, and BBS are included. For all participants we gather PROMs including the selected subscales of SIS[43] described above as well as the SIS visual analogue scale on overall recovery, the Daily Fatigue Impact Scale (D-FIS)[44], a life satisfaction question on contentment with life in general from the Life Satisfactory Questionary[45], and the Hospital Anxiety and Depression Scale (HADS)[46]. At each follow-up visit we also register clinical variables known to influence outcomes and/or changes in these variables since last visit, such as rehabilitation attained, other treatments, vascular risk factors, physical activity, socioeconomic status, infections (including Covid19), and other comorbidities. Moreover, all study participants are invited to repeated neuroimaging with a study-specific protocol at 3-6 months, as well as 1 and 2 years after stroke onset.

To ensure that individuals with the worse outcome/socioeconomic situations are not lost to follow up we collect the data in the following priority order: 1) patients receive questionnaires by mail and come to the hospital for tests, neuroimaging and blood draw; 2) patients receive mail questionnaires and a research nurse conducts a telephone interview; 3) patients receive mail questionnaires only; 4) when none of the above can be implemented, patients will be

For peer review only

followed through national registers including Riksstroke[47], provided they have not chosen to withdraw their participation.

Data retrieval

Data on deaths, and recurrent stroke or other major vascular events, comorbidities, and prescribed drugs over time during the whole study period are obtained from the Swedish Population Register, the National Patient Register, the Prescribed Drugs Register, and the National Register on Cause of Death as described[48]. For events in the Patient Register and the Cause of Death Register we review medical records.

For patients who are unable to participate in our 3-month and/or 1-year follow-up, data on outcomes are obtained from Riksstroke[47], which has data on self-reported outcomes at 3 and 12 months after stroke. The Riksstroke data are transformed to the modified Rankin Scale (mRS) scores 0-2, 3, 4 or 5 using a validated algorithm as described[49]. Data on health and social support are obtained from the National Board of Health and Welfare and data on education and employment status from the Longitudinal Database of Education, Income and Occupation Register (Swedish acronym: LISA for *Longitudinell integrationsdatabas för sjukförsäkrings- och arbetsmarknadsstudie*). Data on management of diabetes and hypertension are obtained from the National Diabetes Register and the Primary Care Registry, respectively.

Stroke subtyping

Ischemic stroke subtyping is performed by a trained physician using validated systems (Trial of Org. 10172 in Acute Stroke Treatment (TOAST) criteria[50], the Causative Classification of Stroke (CCS) system[51] and the Oxfordshire Community Stroke Project (OSCP)

For peer review only

classification[52] for ischemic stroke. Intracerebral bleedings (ICH) are classified using SMASH-U[53].

Blood sampling

A first blood sample is drawn as soon as possible after admission to the stroke unit, and on a subgroup we also draw a sample 7 days after stroke. Thereafter blood samples are drawn at each follow-up visit. On all occasions, venous blood is collected after an overnight fast. Blood is drawn in several tubes and handled at room temperature, whole blood or supernatants, i.e., plasma and serum, are robot aliquoted within 2 hours and stored in freezers at Biobank Väst, a part of the Swedish National Biobank, pending analysis, for details see (Table 2). Blood is also drawn for analyses of total leucocyte count and differential blood count at the Dept of Clinical Chemistry at our hospital.

Blood biomarker analysis

Clinically established blood biomarkers are commonly based on measurements of protein concentrations in plasma or serum, and we and others have previously found plasma proteins with subtype specific patterns[54, 55], plasma protein concentrations associated with recurrent vascular events[56, 57] as well as neurological and functional outcomes[57-60]. Here, planned analyses include multiomics profiling, on the metabolite, protein, RNA and DNA level, utilising the Swedish National infrastructure for state-of-the-art omics atScience for Life Laboratory (SciLifeLab), Uppsala and Stockholm, and the Bioinformatics and Data Centre, Gothenburg.

For peer review only

Table 2 Blood sample handling

Type	Tube(s) and additive	Centrifugation	Storage
Whole blood	With 10%[α] ethylenediaminetetraacetic acid (EDTA)*	NA	-80 °C
EDTA plasma	With 10%[α] EDTA*	2000 g for 10 min	-80 °C
Citrate plasma	With 3.2%[α] natrium citrate*	2500 g for 20 min	-80 °C
Serum	Serum separator tube*	Left to coagulate 30-60 min and spun 2000 g for 10 min	-80 °C
Whole blood	Tempus™ Blood RNA[+]	NA	-20 °C

[α] Volume/volume
* Becton, Dickinson and Company, Becton Drive Franklin Lakes, NJ, USA
[+] Life Technologies, Carlsbad, CA, United States
NA, not applicable

STUDY AIMS

Primary objective

The primary aims are two-fold:

i) Determine trajectories of global as well at specific domains of cognition and neurological function after stroke.

ii) Identify clinical variables, as well as neuroimaging and blood biomarkers, predicting post-stroke cognitive and neurological outcomes.

Secondary objectives

i) Determine trajectories of blood biomarker concentrations after stroke and optimal time points for measurements.

ii) Determine trajectories of upper arm motor function after stroke.

For peer review only

iii) Determine trajectories of self-reported depressive symtoms, fatigue, participation, and quality of life after stroke, and their relations to other outcome measures with focus on cognition.

iv) Identify stroke subtype-specific recovery trajectories.

v) Identify clinical variables, as well as neuroimaging and blood biomarkers, predicting upper arm motor function and self-reported outcomes after stroke.

Organization

The study is conducted with three principal investigators CJ, TT and KJ. The patients are recruited at the stroke-units at the Sahlgrenska University Hospital in Gothenburg. All follow-up invitations and visits are managed by specially trained research nurses. Data analysis will be overseen by the CJ and KJ research groups.

ETHICS AND DISSEMINATION

Ethical approval

This study was approved by the Regional Ethics Review Board in Gothenburg, and the Swedish Ethical Review Authority (REC number 187-17 and 2021-06071-02, respectively, Guarantor: CJ). Written informed consent is obtained from all participants prior to enrolment. For participants who cannot give written concent, consultant consent is obtained from their next-of-kin. Participants may withdraw their consent at any time, and if so, the participants data will be cancelled and counted as missing.

Dissemmination

The protocol is registered at http://www.clinicaltrials.gov, Study ID: NCT05708807.

For peer review only

For peer review only

DISCUSSION

As stated in the Introduction, there is surprisingly little data on trajectories (i.e. repeated measures) of stroke recovery in several domains in itself and even less in combination with biomarkers. This protocol describes the *FIND Stroke Recovery Study*, a unique longitudinal cohort study in stroke with detailed phenotyping and repetitive assessments of outcome measures across multiple domains coupled to biomarker identification. We perform broad cognitive testing, and use NIHSS and mRS, the most used measures of neurological deficits and functional outcome respectively, several motor function measures, essentially in accordance with recently published recommendations[61-63]. We also use several complementary outcome metrics, objectively tested as well as self-reported, to comprehensively capture a broad spectrum of outcomes including different dimensions according to the International Classification of Functioning, Disability and Health. In combination, we collect blood samples for broad biomarker profiling as well as stroke lesion data using advanced neuroimaging. The field of predictive biomarkers in ischemic stroke has not yet reached a conclusive state and even between high quality studies results are to some extent inconsistent[59]. A few ongoing studies have designs similar to FIND and include measurements of neuroimaging and/or blood biomarkers: e.g., STROKE-Cog, R4VaD. OX-CHRONIC, APPLE and DISCOVERY[22-24, 26, 27] including adult stroke survivors. The protocols of these studies have follow-ups allowing for comparisons with some of the cognitive outcomes in our study protocol making external validation of some of our findings possible. However, in comparison to these, FIND is more focused on blood-based protein and RNA biomarkers and notably includes blood sampling at each study visit. In line with our protocol, DISCOVERY[24] also includes MRI and blood-based protein biomarkers whereas STROKE-Cog has a focus on circulating blood biomarkers of immunological responses[23].

For peer review only

R4VaD[26] is mainly focused on dementia after stroke and includes blood biomarkers whereas OX-CHRONIC[22] includes neuroimaging, but not biomarkers.

Cognitive outcome after stroke is of great importance for the patients, their relatives, and care givers. Despite this, trajectories of cognitive recovery after stroke and mechanisms behind the recovery are less studied than motor and other functionalities[64], likely due to the complexity of cognition. Thus, we chose to add several tests assessing similar domains to capture different aspects and nuances of cognition. We utilize the commonly used MoCA and add tests of different specific cognitive domains such as processing speed (e.g. TMT-A and CWT 1-2), attention (e.g. RSCT), executive functions (e.g. TMT-B and CWT 3), memory (10-word test from Repeatable Battery), and language and verbal fluency (BNT and FAS). These tests capture specific cognitive domains as well as domains with potentials for a more global influence on cognitive function such as executive functions, complex attention and information processing speed. The latter test are included because we believe that these are understudied in stroke. Moreover, such tests might be more sensitive to monitoring impairments than domain-specific measures in long term follow-ups after stroke. We include additional cognitive tests for participants <55 years at inclusion than for those >55 years. The rationale for a more detailed cognitive mapping in younger participants is their longer expected life-time meaning many years of productive life living with impairments in combination with the generally higher expectations and demands on cognitive function. Moreover, we know from experience that the ability of stroke survivors to perform a large battery of cognitive tests concentrated to one day is better in the young than in the older population. There is another ongoing prospective study of trajectories of cognitive function after stroke, ODYSSEY[25] focusing on stroke in young adults. However, this study is not focused on cognition and does not include blood biomarkers. To complement the objective cognitive tests, we also include self-reported cognitive function (eg. SIS). Simultaneous to

For peer review only - http://bmjopen.bmj.com/site/about/guidelines.xhtml

assessments of cognitive outcome, we study neurological outcomes with NIHSS total and subscale scores as well as finer grained assessments of upper arm motor function. We also assess functional outcome by the commonly used mRS and include PROMs (e.g. domains of SIS) to complement our objective functionality measures. Moreover, as mRS is only moderately correlated to quality of life[65], we include a question on life satisfaction.

Stroke is associated with increased rates of depression and a previous study has found about one third of stroke patients to be affected by post-stroke depression[66]. A correlation between depressive symptoms (HADS-D >7) and performance in cognitive tests both on the global and domain specific level has been reported[67]. Also, depressive symptoms measured as HAD score >4 have been found to correlate to self-reported memory and thinking problems (using SIS)[68]. Because depression and cognitive impairment seem to be connected and reciprocally influential in stroke survivors, we are assessing mental health trajectories in our cohort. To enable comparison of our results with those from as many studies as possible we decided to include the widely used HADS which also includes anxiety.

Fatigue, a common symptom after stroke, can also add to cognitive dysfunction. Therefore, we included D-FIS to measure the impact of fatigue on cognitive recovery in this cohort. D-FIS is highly correlated to the more extensive Fatigue Impact Scale[69] but less time consuming for the patient.

Etiologic subtype of ischemic stroke has been reported to associate with cognitive outcome[15], but also with other aspects of post-stroke outcome such as persisting neurological deficits[70]. The study protocol presented here allows for subtyping of all ischemic strokes according to two validated systems and subtype-specific analysis of outcome measures. In line with this we also have the possibility to classify the haemorrhagic stroke's etiology into structural vascular lesions, medication, amyloid angiopathy, systemic disease, hypertension, or undetermined

For peer review only

using the SMASH-U[53] classification system and to correlate the subtypes to biomarkers and outcome measures.

Acute imaging in stroke using CT and/or MRI aims primarily at identification of location and size of a lesion, exclusion of haemorrhage prior to treatment, differentiation between irreversibly affected brain tissue and tissue at risk, as well as identification of stenosis or occlusion in supplying vessels. We also perform neuroimaging with MRI at inclusion and perform detailed mapping of location and size of the lesion. Imaging can, however, also provide insights on pre-stroke status of the brain as well as poststroke tissue integrity within the lesion and in normal appearing cerebral tissue. Pre-existing disease markers, such as white matter lesions, atrophy, microinfarcts and co-morbidity of other aetiology, and post-stroke tissue integrity markers, such as metabolic, diffusion, and perfusion metrics, aide us to monitor disease progression and to target outcome prediction[36]. We also perform MRI at three time points post-stroke in order to assess for instance white matter condition.

Strengths and limitations

The strengths of the present study include time-resolved data of post-stroke recovery in a sample of consecutively recruited and well-characterized stroke patients of all ages. Moreover, the follow-up includes a comprehensive set of detailed outcome metrics with focus on cognitive and neurological function with global as well as a domain specific measures. Taken together with our broad, repetitive biomarker profiling, in blood and using advanced MRI, this allows for exploration of biomarkers for cognitive, neurological and functional outcomes as well as fatigue and depressive symptoms after stroke. Notably, this study is in accordance with guidelines for stroke outcome studies, that has been published after initiation of the present protocol[61]. Further strengths lie in the design of our protocol, which was influenced by a referendum with study participants, research nurses and representatives from the local stroke patient association. Nonetheless, our study has important limitations. A

For peer review only

limitation is heterogeneity in the time point of sample collection in the acute phase. For practical reasons it is not always possible to include participants withing 24 hours after symptom onset. We prioritize to be able to include as many study participants as possible and document the time of sampling to enable analysis of impact of the sampling time on the data. Another limitation, shared with most long-term follow-up studies after strokes, is that persons suffering the most severe strokes will be underrepresented in the data, skewing results towards mild strokes. This is due to both lower inclusion rate and higher likelihood for withdrawal from the study follow ups in severe stroke patients. To our advantage, we opt for telephone follow-ups when a physical visit is not possible. Moreover, very few study participants are fully lost as even though they will not come to follow-ups we can still follow them through national registers, such as the Riksstroke Register, and the National Patient Register with consent from the participant. Also, due to the COVID-19 pandemic there will be a period of several months with lower inclusion rate and modified follow-up visits. A third limitation is that several cognitive tests used by us (and others) rely on language and the cognitive function measures of partcipants with aphasia may be affected. We have in line with the previously mentioned R4VaD[26] study decided to include participants with aphasia and encourage them to complete the study protocol, if possible, while other ongoing studies has decided to exclude patients with aphasia[23].

Significance / Conclusion

The *FIND Stroke Recovery study* will provide an unprecedented opportunity to inform on the time course of recovery coupled to detailed biomarker analyses (imaging, multiomic profiling of protein, RNA and DNA) after stroke. The gained knowledge from this project will be helpful for designing future studies on biomarkers and interventions targeting stroke recovery. Moreover, this study will provide an opportunity to find parts of the puzzle of extent and rate

For peer review only

of recovery for an individual. Hopefully, together with other studies in the field, it will lead to better information on prognosis to patients and their loved ones.

DECLARATIONS

Funding

This work is supported by the Swedish Research Council (2021-01114), the Swedish state under the agreement between the Swedish government and the county councils, the ALF-agreement (ALFGBG-965328, ALFGBG-965417, ALFGBG-966177, and ALFGBG-942664), the Swedish Heart and Lung Foundation (20220184), and the King Gustaf V:s and Queen Victoria's Foundation.

Contributorship statement

CB wrote the manuscript; SK, TMS, KSS, MAM, HS, IBB, OJ and DÅ contributed valuable expertise to study outline; KJ, TT and CJ conceived the study and are co-PIs of the study. All authors edited the manuscript and have approved the final version. CJ is the guarantor of the study.

Competing interests

Authors report no competing interests.

For peer review only

REFERENCES

1. Global, regional, and national burden of neurological disorders, 1990-2016: a systematic analysis for the Global Burden of Disease Study 2016. *Lancet Neurol*. 2019; 18: 459-80.
2. Collaborators GBDS. Global, regional, and national burden of stroke and its risk factors, 1990-2019: a systematic analysis for the Global Burden of Disease Study 2019. *Lancet Neurol*. 2021; 20: 795-820.
3. Redfors P, Isaksén D, Lappas G, et al. Living alone predicts mortality in patients with ischemic stroke before 70 years of age: a long-term prospective follow-up study. *BMC neurology*. 2016; 16: 80.
4. Alawieh A, Zhao J and Feng W. Factors affecting post-stroke motor recovery: Implications on neurotherapy after brain injury. *Behavioural brain research*. 2018; 340: 94-101.
5. Lindgren A and Maguire J. Stroke Recovery Genetics. *Stroke*. 2016; 47: 2427-34.
6. Lee JM, Fernandez-Cadenas I and Lindgren AG. Using human genetics to understand mechanisms in ischemic stroke outcome: from early brain injury to long-term recovery. *Stroke*. 2021; 52: 3013-24.
7. Bernhardt J, Hayward KS, Kwakkel G, et al. Agreed definitions and a shared vision for new standards in stroke recovery research: The Stroke Recovery and Rehabilitation Roundtable taskforce. *International Journal of Stroke*. 2017; 12: 444-50.
8. Toschke AM, Tilling K, Cox AM, Rudd AG, Heuschmann PU and Wolfe CD. Patient-specific recovery patterns over time measured by dependence in activities of daily living after stroke and post-stroke care: the South London Stroke Register (SLSR). *Eur J Neurol*. 2010; 17: 219-25.
9. Lee KB, Lim SH, Kim KH, et al. Six-month functional recovery of stroke patients: a multi-time-point study. *International Journal of Rehabilitation Research*. 2015; 38: 173-80.
10. Verheyden G, Nieuwboer A, De Wit L, et al. Time course of trunk, arm, leg, and functional recovery after ischemic stroke. *Neurorehabilitation and Neural Repair*. 2008; 22: 173-9.
11. Chye A, Hackett ML, Hankey GJ, et al. Repeated measures of modified Rankin scale scores to assess functional recovery from stroke: AFFINITY study findings. *Journal of the American Heart Association*. 2022; 11: e025425.
12. Demeyere N, Riddoch MJ, Slavkova ED, et al. Domain-specific versus generalized cognitive screening in acute stroke. *Journal of neurology*. 2016; 263: 306-15.
13. Webb SS, Hobden G, Roberts R, Chiu EG, King S and Demeyere N. Validation of the UK English Oxford cognitive screen-plus in sub-acute and chronic stroke survivors. *Eur Stroke J*. 2022; 7: 476-86.
14. Jaillard A, Naegele B, Trabucco-Miguel S, LeBas JF and Hommel M. Hidden dysfunctioning in subacute stroke. *Stroke*. 2009; 40: 2473-9.
15. Saa JP, Tse T, Baum CM, et al. Cognitive Recovery After Stroke: A Meta-analysis and Metaregression of Intervention and Cohort Studies. *Neurorehabil Neural Repair*. 2021; 35: 585-600.
16. del Ser T, Barba R, Morin MM, et al. Evolution of cognitive impairment after stroke and risk factors for delayed progression. *Stroke*. 2005; 36: 2670-5.
17. Lo JW, Crawford JD, Desmond DW, et al. Long-term cognitive decline after stroke: an individual participant data meta-analysis. *Stroke*. 2022; 53: 1318-27.
18. Hochstenbach JB, den Otter R and Mulder TW. Cognitive recovery after stroke: a 2-year follow-up. *Arch Phys Med Rehabil*. 2003; 84: 1499-504.
19. Turunen KEA, Laari SPK, Kauranen TV, et al. Domain-specific cognitive recovery after first-ever stroke: a 2-year follow-up. *J Int Neuropsychol Soc*. 2018; 24: 117-27.
20. Demeyere N, Sun S, Milosevich E and Vancleef K. Post-stroke cognition with the Oxford Cognitive Screen vs Montreal Cognitive Assessment: a multi-site randomized controlled study (OCS-CARE) [version 1; peer review: 1 approved, 1 approved with reservations]. *Health Open Research*. 2019; 1.
21. Patel M, Coshall C, Rudd AG and Wolfe CD. Natural history of cognitive impairment after stroke and factors associated with its recovery. *Clin Rehabil*. 2003; 17: 158-66.

For peer review only

22. Demeyere N, Williams OA, Milosevich E, et al. Long-term psychological consequences of stroke (OX-CHRONIC): A longitudinal study of cognition in relation to mood and fatigue after stroke: Protocol. *Eur Stroke J*. 2021; 6: 428-37.
23. Drag LL, Mlynash M, Nassar H, et al. A longitudinal study of the post-stroke immune response and cognitive functioning: the StrokeCog study protocol. *BMC neurology*. 2020; 20: 313.
24. Rost NS, Meschia JF, Gottesman R, Wruck L, Helmer K and Greenberg SM. Cognitive Impairment and Dementia After Stroke: Design and Rationale for the DISCOVERY Study. *Stroke*. 2021; 52: e499-e516.
25. Arntz RM, van Alebeek ME, Synhaeve NE, et al. Observational Dutch Young Symptomatic StrokE studY (ODYSSEY): study rationale and protocol of a multicentre prospective cohort study. *BMC neurology*. 2014; 14: 55.
26. Wardlaw JM, Doubal F, Brown R, et al. Rates, risks and routes to reduce vascular dementia (R4vad), a UK-wide multicentre prospective observational cohort study of cognition after stroke: protocol. *Eur Stroke J*. 2021; 6: 89-101.
27. Quinn TJ, Taylor-Rowan M, Elliott E, et al. Research protocol - assessing post-stroke psychology longitudinal evaluation (APPLE) study: a prospective cohort study in stroke. *Cerebral circulation - cognition and behavior*. 2022; 3: 100042.
28. Boyd LA, Hayward KS, Ward NS, et al. Biomarkers of stroke recovery: Consensus-Based core recommendations from the Stroke Recovery and Rehabilitation Roundtable. *Neurorehabil Neural Repair*. 2017; 31: 864-76.
29. Kamtchum-Tatuene J and Jickling GC. Blood Biomarkers for Stroke Diagnosis and Management. *Neuromolecular Med*. 2019; 21: 344-68.
30. Klil-Drori S, Phillips N, Fernandez A, Solomon S, Klil-Drori AJ and Chertkow H. Evaluation of a Telephone Version for the Montreal Cognitive Assessment: Establishing a Cutoff for Normative Data From a Cross-Sectional Study. *Journal of geriatric psychiatry and neurology*. 2022; 35: 374-81.
31. Katz MJ, Wang C, Nester CO, et al. T-MoCA: A valid phone screen for cognitive impairment in diverse community samples. *Alzheimer's & dementia (Amsterdam, Netherlands)*. 2021; 13: e12144.
32. Lai Y, Zhao M, Jiang C, et al. Validation of T-MoCA in the Screening of Mild Cognitive Impairment in Chinese Patients With Atrial Fibrillation. *Frontiers in cardiovascular medicine*. 2022; 9: 896846.
33. Mehrholz J, Wagner K, Rutte K, Meissner D and Pohl M. Predictive validity and responsiveness of the functional ambulation category in hemiparetic patients after stroke. *Arch Phys Med Rehabil*. 2007; 88: 1314-9.
34. Berg KO, Wood-Dauphinee SL, Williams JI and Maki B. Measuring balance in the elderly: validation of an instrument. *Canadian journal of public health = Revue canadienne de sante publique*. 1992; 83 Suppl 2: S7-11.
35. Nasreddine ZS, Phillips NA, Bédirian V, et al. The Montreal Cognitive Assessment, MoCA: a brief screening tool for mild cognitive impairment. *J Am Geriatr Soc*. 2005; 53: 695-9.
36. Sagnier S and Sibon I. The new insights into human brain imaging after stroke. *Journal of neuroscience research*. 2022; 100: 1171-81.
37. Reitan RM. The relation of the trail making test to organic brain damage. *Journal of consulting psychology*. 1955; 19: 393-4.
38. Lowery N, Ragland JD, Gur RC, Gur RE and Moberg PJ. Normative data for the symbol cancellation test in young healthy adults. *Appl Neuropsychol*. 2004; 11: 218-21.
39. Delis D KE, Kramer J. . D-KEFS, Delis-Kaplan Executive Function System. *San Antonio, TX: The Psychological Corporation; 2001*. 2001.
40. Randolph C. Repeatable Battery for the Assessment of Neuropsychological Status (RBANS). *Psychological Corporation San Antonio, TX, 1998.*
41. Strauss E SE, Spreen O. *A compendium of neuropsychological tests: administration, norms, and commentary, 3rd edn.*: Oxford, NY: Oxford University Press, 2006.

For peer review only

42. Mack WJ, Freed DM, Williams BW and Henderson VW. Boston Naming Test: shortened versions for use in Alzheimer's disease. *J Gerontol*. 1992; 47: P154-8.
43. Duncan PW, Bode RK, Min Lai S, Perera S and Glycine Antagonist in Neuroprotection Americans I. Rasch analysis of a new stroke-specific outcome scale: the Stroke Impact Scale. *Arch Phys Med Rehabil*. 2003; 84: 950-63.
44. Fisk JD and Doble SE. Construction and validation of a fatigue impact scale for daily administration (D-FIS). *Quality of Life Research*. 2002; 11: 263-72.
45. Fugl-Meyer AR, Bränholm I-B and Fugl-Meyer KS. Happiness and domain-specific life satisfaction in adult northern Swedes. *Clinical Rehabilitation*. 1991; 5: 25-33.
46. Zigmond AS and Snaith RP. The hospital anxiety and depression scale. *Acta psychiatrica Scandinavica*. 1983; 67: 361-70.
47. Riksstroke.org.2021. RIKSSTROKE The Swedish Stroke Register.
48. Jood K, Ladenvall C, Rosengren A, Blomstrand C and Jern C. Family history in ischemic stroke before 70 years of age: the Sahlgrenska Academy Study on Ischemic Stroke. *Stroke*. 2005; 36: 1383-7.
49. Eriksson M, Appelros P, Norrving B, Terént A and Stegmayr B. Assessment of Functional Outcome in a National Quality Register for Acute Stroke. *Stroke*. 2007; 38: 1384-6.
50. Adams HP, Jr., Bendixen BH, Kappelle LJ, et al. Classification of subtype of acute ischemic stroke. Definitions for use in a multicenter clinical trial. TOAST. Trial of Org 10172 in Acute Stroke Treatment. *Stroke*. 1993; 24: 35-41.
51. Ay H, Furie KL, Singhal A, Smith WS, Sorensen AG and Koroshetz WJ. An evidence-based causative classification system for acute ischemic stroke. *Ann Neurol*. 2005; 58: 688-97.
52. Bamford J, Sandercock P, Dennis M, Burn J and Warlow C. Classification and natural history of clinically identifiable subtypes of cerebral infarction. *Lancet*. 1991; 337: 1521-6.
53. Meretoja A, Strbian D, Putaala J, et al. SMASH-U: a proposal for etiologic classification of intracerebral hemorrhage. *Stroke*. 2012; 43: 2592-7.
54. Stanne TM, Angerfors A, Andersson B, Brännmark C, Holmegaard L and Jern C. Longitudinal Study Reveals Long-Term Proinflammatory Proteomic Signature After Ischemic Stroke Across Subtypes. *Stroke*. 2022; 53: 2847-58.
55. Kang YH, Park MG, Noh KH, et al. Low serum TNF-related apoptosis-inducing ligand (TRAIL) levels are associated with acute ischemic stroke severity. *Atherosclerosis*. 2015; 240: 228-33.
56. Pedersen A, Redfors P, Lundberg L, et al. Haemostatic biomarkers are associated with long-term recurrent vascular events after ischaemic stroke. *Thromb Haemost*. 2016; 116: 537-43.
57. Bao Q, Zhang J, Wu X, et al. Clinical Significance of Plasma D-Dimer and Fibrinogen in Outcomes after Stroke: A Systematic Review and Meta-Analysis. *Cerebrovasc Dis*. 2022: 1-26.
58. Pedersen A, Stanne TM, Redfors P, et al. Fibrinogen concentrations predict long-term cognitive outcome in young ischemic stroke patients. *Res Pract Thromb Haemost*. 2018; 2: 339-46.
59. Montellano FA, Ungethüm K, Ramiro L, et al. Role of Blood-Based Biomarkers in Ischemic Stroke Prognosis: A Systematic Review. *Stroke*. 2021; 52: 543-51.
60. Lagging C, Klasson S, Pedersen A, et al. Investigation of 91 proteins implicated in neurobiological processes identifies multiple candidate plasma biomarkers of stroke outcome. *Sci Rep*. 2022; 12: 20080.
61. Lindgren AG, Braun RG, Juhl Majersik J, et al. International stroke genetics consortium recommendations for studies of genetics of stroke outcome and recovery. *International Journal of Stroke*. 2022; 17: 260-8.
62. Prange-Lasonder GB, Alt Murphy M, Lamers I, et al. European evidence-based recommendations for clinical assessment of upper limb in neurorehabilitation (CAULIN): data synthesis from systematic reviews, clinical practice guidelines and expert consensus. *J Neuroeng Rehabil*. 2021; 18: 162.
63. Kwakkel G, Lannin NA, Borschmann K, et al. Standardized measurement of sensorimotor recovery in stroke trials: Consensus-based core recommendations from the Stroke Recovery and Rehabilitation Roundtable. *Neurorehabil Neural Repair*. 2017; 31: 784-92.

For peer review only - http://bmjopen.bmj.com/site/about/guidelines.xhtml

64. McDonald MW, Black SE, Copland DA, et al. Cognition in stroke rehabilitation and recovery research: Consensus-based core recommendations from the second Stroke Recovery and Rehabilitation Roundtable. *International Journal of Stroke*. 2019; 14: 774-82.
65. Skoglund E, Westerlind E, Persson HC and Sunnerhagen KS. Self-perceived impact of stroke: A longitudinal comparison between one and five years post-stroke. *Journal of rehabilitation medicine*. 2019; 51: 660-4.
66. Hommel M, Carey L and Jaillard A. Depression: cognition relations after stroke. *International journal of stroke : official journal of the International Stroke Society*. 2015; 10: 893-6.
67. Williams OA and Demeyere N. Association of Depression and Anxiety With Cognitive Impairment 6 Months After Stroke. *Neurology*. 2021; 96: e1966-e74.
68. Ytterberg C, Cegrell L, von Koch L and Wiklander M. Depression symptoms 6 years after stroke are associated with higher perceived impact of stroke, limitations in ADL and restricted participation. *Sci Rep*. 2022; 12: 7816.
69. Pedersen A, Almkvist E, Holmegaard L, et al. Fatigue 7 years post-stroke: Predictors and correlated features. *Acta Neurol Scand*. 2022; 146: 295-303.
70. Pinto A, Tuttolomondo A, Di Raimondo D, Fernandez P and Licata G. Risk factors profile and clinical outcome of ischemic stroke patients admitted in a Department of Internal Medicine and classified by TOAST classification. *International angiology : a journal of the International Union of Angiology*. 2006; 25: 261-7.

Acknowledgements

The authors thank research nurses Susanne Nilsson, Ingrid Eriksson, Virginia Villarreal Calvo, Magdalena Hagelberg, and Matilda Errind Arvgård for their excellent work and assistance in recruitment of study participants and for conducting follow-up visits. We also thank the physiotherapists at the stroke unit performing the motor assessments. Furthermore, we thank the stroke survivors and their kin for generously donating their time to this study. In addition, we thank the Biobank Väst, Bioinformatics and Data Centre, Gothenburg, and SciLifeLab**.**

Figure legends

Figure 1 Overview of study protocol

For peer review only

* Performed at all visits except for inclusion. ** Only at inclusion. *** Not conducted at inclusion or at 6 months follow-up to prevent repetition bias. ¨ Performed at all visits except at 1 year. Only performed at 3 months follow-up. $ for participants < 55 years old at index stroke. ø If aphasia at inclusion. ^ If deficits at inclusion. These fixed time points are complimented by register studies that constantly monitor recurrent stroke and other vascular events, medication, and deaths.

Abbreviations: Daily Fatigue Impact Scale (D-FIS), Hospital Anxiety and Depression Scale (HADS), Life Satisfactory Questionary (Lisat11), Stroke Impact Scale (SIS), Modified Rankin Scale (mRS), National Institutes of Health Stroke Scale (NIHSS), Verbal Fluency Test (FAS), Color-Word Interference test (CWT), Trailmaking Test (TMT), Random Shape Cancellation Test (RSCT), Repeatable Battery for the Assessment of Neuropsychological Status (RBANS), Montreal Cognitive Assessment (MoCA), the 15-Item Boston Naming Test (BNT), Addenbrooke's Cognitive Examination-Revised test (ACE-R), Berg Balance Scale (BBS), functional Ambulation Category (FAC), Shoulder Abduction and Finger Extension (SAFE), Fugl–Meyer Assessment of Upper Extremity (FMA).

For peer review only

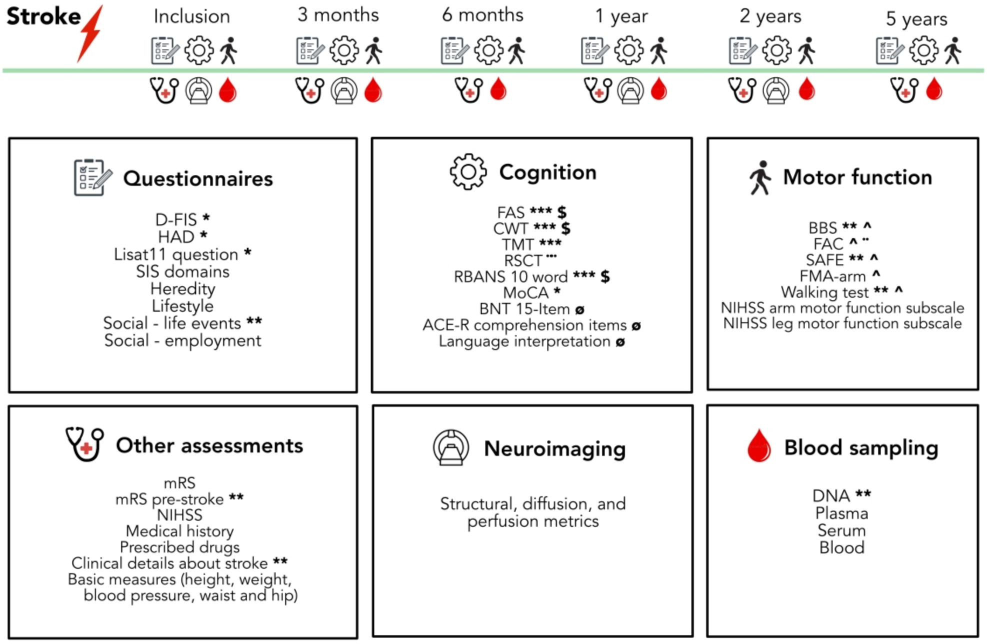

Figure 1 Overview of study protocol
* Performed at all visits except for inclusion. ** Only at inclusion. *** Not conducted at inclusion or at 6 months follow-up to prevent repetition bias. ¨ Performed at all visits except at 1 year. ˙ ˙ ˙ Only performed at 3 months follow-up. $ for participants < 55 years old at index stroke. ø If aphasia at inclusion. ^ If deficits at inclusion. These fixed time points are complimented by register studies that constantly monitor recurrent stroke and other vascular events, medication, and deaths.

Abbreviations: Daily Fatigue Impact Scale (D-FIS), Hospital Anxiety and Depression Scale (HADS), Life Satisfactory Questionary (Lisat11), Stroke Impact Scale (SIS), Modified Rankin Scale (mRS), National Institutes of Health Stroke Scale (NIHSS), Verbal Fluency Test (FAS), Color-Word Interference test (CWT), Trailmaking Test (TMT), Random Shape Cancellation Test (RSCT), Repeatable Battery for the Assessment of Neuropsychological Status (RBANS), Montreal Cognitive Assessment (MoCA), the 15-Item Boston Naming Test (BNT), Addenbrooke's Cognitive Examination-Revised test (ACE-R), Berg Balance Scale (BBS), functional Ambulation Category (FAC), Shoulder Abduction and Finger Extension (SAFE), Fugl–Meyer Assessment of Upper Extremity (FMA).

755x534mm (118 x 118 DPI)

Supplemental Table 1 Original protocol

Parameter	Details	Baseline	Follow-up 3, 6, 12 months, 2 and 5 years
Neurological impairment	NIHSS	X	X
Functional outcome	mRS	X	X
Cognitive function	MoCA, BNIS[a], Functional Ambulation Category, 15-Item Boston Naming Test*, Addenbrooke's Cognitive Examination-Revised test*	X	X
Motor function	FMA*	X	X
Cognitive function	Mesulam A and B, Trailmaking Test A, B and D[b], 10-word test from Repeatable Battery Mesulams test, modified Ashworth Scale for the Assessment of Neuropsychological Status[ǂ], FAS[ǂc], Color-Word Interference test[ǂ], Letter-Number Sequencing Subtest from WAIS-VI[d],		X
Motor function	SAFE, Berg Balance Scale, 10m walking test	X	
Pre-stroke functional status, cognitive function, education and profession	SIS[e], IQCODE[f]	X	
Pre-stroke infection or major life event	Questionnaire	X	
Depression	HAD, PHQ-9[g], MADRS[h]		X
Fatigue	D-FIS		X
Sleepiness	ESS[i]		X
Quality of Life	Lisat 11[j]		X
Working and social situation	Questionnaire	X	X
Medical history, medication and rehab	Questionnaire	X	X
Cardiovascular risk factors	Questionnaire	X	X
Stroke-specific composite disability score	SIS[e]		X
Venous blood sampling	For protein biomarker profiling and extraction of genomic RNA	X	X

For peer review only

Stroke subtype	TOAST, OSCP and CSS	X

* If full function at baseline no further testing
ǂ Only patients <55 years at inclusion
[a] The Barrow Neurological Institute screen[1] for higher cerebral functions
[b] Trail making test D[2]
[c] Verbal Fluency semantic test FAS
[d] Letter-Number Sequencing Subtest from WAIS-VI[3]
[e] Stroke Impact Scale, full test, some domains still included (see methods section)
[f] Informant Questionnaire on Cognitive Decline in the Elderly[4]
[g] PHQ-9[5]
[h] Montgomery Åsberg Depression Rating Scale[6]
[i] Epworth Sleepiness Scale[7]
[j] Life Satisfaction Questionnaire[8]

REFERENCES

1. Prigatano GP. BNI Screen for higher cerebral functions: Rationale and initial validation. *BnI Quarterly*. 1991; 7: 2-9.
2. Llinàs-Reglà J, Vilalta-Franch J, López-Pousa S, Calvó-Perxas L, Torrents Rodas D and Garre-Olmo J. The Trail Making Test:Association With Other Neuropsychological Measures and Normative Values for Adults Aged 55 Years and Older From a Spanish-Speaking Population-Based Sample. *Assessment*. 2017; 24: 183-96.
3. Wechsler D. Wechsler adult intelligence scale–Fourth Edition (WAIS–IV). *San Antonio, TX: NCS Pearson*. 2008; 22: 1.
4. Jorm AF. The Informant Questionnaire on Cognitive Decline in the Elderly (IQCODE): a review. *International Psychogeriatrics*. 2004; 16: 275-93.
5. Kroenke K, Spitzer RL and Williams JB. The PHQ-9: validity of a brief depression severity measure. *Journal of general internal medicine*. 2001; 16: 606-13.
6. Montgomery SA and Asberg M. A new depression scale designed to be sensitive to change. *The British journal of psychiatry : the journal of mental science*. 1979; 134: 382-9.
7. Johns MW. A new method for measuring daytime sleepiness: the Epworth sleepiness scale. *Sleep*. 1991; 14: 540-5.
8. Fugl-Meyer AR, Bränholm I-B and Fugl-Meyer KS. Happiness and domain-specific life satisfaction in adult northern Swedes. *Clinical Rehabilitation*. 1991; 5: 25-33.

For peer review only

STROBE Statement—Checklist of items that should be included in reports of ***cohort studies***

	Item No	Recommendation	Page No
**Title and abstract**	1	(*a*) Indicate the study's design with a commonly used term in the title or the abstract	1
		(*b*) Provide in the abstract an informative and balanced summary of what was done and what was found	3-4
**Introduction**			
Background/rationale	2	Explain the scientific background and rationale for the investigation being reported	5-7
Objectives	3	State specific objectives, including any prespecified hypotheses	15-16
**Methods**			
Study design	4	Present key elements of study design early in the paper	7
Setting	5	Describe the setting, locations, and relevant dates, including periods of recruitment, exposure, follow-up, and data collection	7
Participants	6	(*a*) Give the eligibility criteria, and the sources and methods of selection of participants. Describe methods of follow-up	7-8
		(*b*) For matched studies, give matching criteria and number of exposed and unexposed	Not applicable
Variables	7	Clearly define all outcomes, exposures, predictors, potential confounders, and effect modifiers. Give diagnostic criteria, if applicable	9-14
Data sources/ measurement	8*	For each variable of interest, give sources of data and details of methods of assessment (measurement). Describe comparability of assessment methods if there is more than one group	7-14
Bias	9	Describe any efforts to address potential sources of bias	12-13
Study size	10	Explain how the study size was arrived at	Not applicable
Quantitative variables	11	Explain how quantitative variables were handled in the analyses. If applicable, describe which groupings were chosen and why	Not applicable
Statistical methods	12	(*a*) Describe all statistical methods, including those used to control for confounding	Not applicable
		(*b*) Describe any methods used to examine subgroups and interactions	
		(*c*) Explain how missing data were addressed	
		(*d*) If applicable, explain how loss to follow-up was addressed	
		(*e*) Describe any sensitivity analyses	
**Results**			
Participants	13*	(a) Report numbers of individuals at each stage of study—eg numbers potentially eligible, examined for eligibility, confirmed eligible, included in the study, completing follow-up, and analysed	Not applicable
		(b) Give reasons for non-participation at each stage	
		(c) Consider use of a flow diagram	
Descriptive data	14*	(a) Give characteristics of study participants (eg demographic, clinical, social) and information on exposures and potential confounders	Not applicable
		(b) Indicate number of participants with missing data for each variable of interest	
		(c) Summarise follow-up time (eg, average and total amount)	
Outcome data	15*	Report numbers of outcome events or summary measures over time	Not applicable

For peer review only - http://bmjopen.bmj.com/site/about/guidelines.xhtml

Main results	16	(*a*) Give unadjusted estimates and, if applicable, confounder-adjusted estimates and their precision (eg, 95% confidence interval). Make clear which confounders were adjusted for and why they were included<br>(*b*) Report category boundaries when continuous variables were categorized<br>(*c*) If relevant, consider translating estimates of relative risk into absolute risk for a meaningful time period	Not applicable
Other analyses	17	Report other analyses done—eg analyses of subgroups and interactions, and sensitivity analyses	Not applicable
**Discussion**			
Key results	18	Summarise key results with reference to study objectives	Not applicable
Limitations	19	Discuss limitations of the study, taking into account sources of potential bias or imprecision. Discuss both direction and magnitude of any potential bias	17-21
Interpretation	20	Give a cautious overall interpretation of results considering objectives, limitations, multiplicity of analyses, results from similar studies, and other relevant evidence	Not applicable
Generalisability	21	Discuss the generalisability (external validity) of the study results	Not applicable
**Other information**			
Funding	22	Give the source of funding and the role of the funders for the present study and, if applicable, for the original study on which the present article is based	21-22

*Give information separately for exposed and unexposed groups.

Note: An Explanation and Elaboration article discusses each checklist item and gives methodological background and published examples of transparent reporting. The STROBE checklist is best used in conjunction with this article (freely available on the Web sites of PLoS Medicine at http://www.plosmedicine.org/, Annals of Internal Medicine at http://www.annals.org/, and Epidemiology at http://www.epidem.com/). Information on the STROBE Initiative is available at http://www.strobe-statement.org.

For peer review only
